# Enhanced performance of glycerol electro-oxidation in alkaline media using bimetallic Au–Cu NPs supported by MWCNTs and reducible metal oxides

**DOI:** 10.3389/fchem.2023.1165303

**Published:** 2023-07-03

**Authors:** Josefina de Gyves, Luis G. Molina-Ruiz, Erik Rutz-López, Ana Lilia Ocampo, Alejandro Gutiérrez-Sánchez, Nadia M. Munguía-Acevedo, Frida Peña-Medina, Vicente Esquivel-Peña

**Affiliations:** Departamento de Química Analítica, Facultad de Química, Universidad Nacional Autónoma de México, Ciudad de México, México

**Keywords:** catalytic electroactivity, cyclic voltammetry, glycerol electro-oxidation, oxidation state influence, hybrid catalysts, microwave assisted, cerium oxide, titanium oxide

## Abstract

Electrochemical technologies for valorizing glycerol, a byproduct of biodiesel production, into electric energy and value-added chemical products continue to be technologically and economically challenging. In this field, an ongoing challenge is developing more active, stable, and low-cost heterogeneous catalysts for the glycerol electro-oxidation reaction (GlyEOR). This paper reports the influence of the preparation procedure, which involves intermatrix synthesis (Cu and Au NPs), followed by galvanic displacement (Cu–Au NPs) in previously functionalized multi-walled carbon nanotubes (MWCNTs). It also discusses the role of the supports, CeO_2_ NPs, and TiO_2_ NPs, obtained by a hydrothermal microwave-assisted procedure, on the electroactivity of a hybrid bimetallic Cu–Au/MWCNT/MO_2_ catalyst in the GlyEOR in alkaline media. The electrocatalytic behavior was studied and discussed in terms of structure, composition, and electroactivity of the synthesized materials, which were determined by Fourier-transform infrared spectroscopy (FTIR), flame atomic absorption spectroscopy (FAAS), transmission electron microscopy (TEM), scanning transmission electron microscopy (STEM), X-ray photoelectronic spectroscopy (XPS), and cyclic voltammetry (CV). In addition, the role of the oxidation states of Cu and Au in the as-prepared catalysts (Cu/MWCNT, Au/MWCNT, Cu–Au/MWCNT, Cu–Au/MWCNT–CeO_2_, and Cu–Au/MWCNT–TiO_2_) was demonstrated. It was concluded that the preparation method of metal NPs for the controlled formation of the most catalytically active oxidation states of Cu and Au, together with the presence of a conductive and oxophilic microenvironment provided by carbon nanotubes and facile reducible oxides in optimized compositions, allows for an increase in the catalytic performance of synthesized catalysts in the GlyEOR.

## 1 Introduction

Bio-based processes to produce chemicals, fuels, and daily consumption materials replacing fossil fuels have experienced vertiginous demand in the last two decades, mainly because of climate change concerns and increasing interest in the sustainable use of natural resources. The development of more efficient and sustainable fuel cells based on polymer electrolyte membranes (PEMFC) is a significant challenge in the field of energy conversion systems. The major drawbacks are high cost and limited activity of the catalyst. Currently, much research in the PEMFC field has focused on the use of biofuels obtained from biomass. The industrial process to produce biodiesel consists of the transesterification of vegetable oils or animal fat with alcohol. In the biofuel industry, during bioethanol and biodiesel production, glycerol is obtained as a byproduct that can be fed directly to a fuel cell. Furthermore, in addition to allowing energy production due to the catalytic oxidation of hydroxyl groups, the glycerol molecule also presents high functionality for developing new processes for producing fine chemical compounds. This conversion of glycerol has been investigated chemically and, to a lesser degree, by electrochemical methods, both in acidic and alkaline media. Previous reports state that the chemical oxidation of glycerol proceeds slowly in the presence of oxidizing agents and heterogeneous catalysts, which result in profitable and eco-friendly processes. The most frequently used catalysts for the selective oxidation of glycerol to fine chemicals are supported mono-, bi-, and multi-metallic catalysts, usually based on Pt and Pd, owing to their high activity and stability (Coutanceau et al., 2019). In addition, Au appears to be an interesting option for the electro-oxidation of glycerol in alkaline media (Kahyaoglu et al., 1984; Beden et al., 1987; Waszczuk et al., 2001) because it is more abundant and, hence, less expensive than other noble metals. Moreover, Au is recognized to be more selective, stable, and less self-poisoned by strongly adsorbed byproducts than Pt and Pd (Talebian-Kiakalaieh et al., 2018). In recent years, adding a second metal to a monometallic catalyst has been proved to enhance not only the catalytic activity but also the stability and/or selectivity of a particular product (Sankar et al., 2012). As mentioned by Alonso et al. (2012), the promotion of the catalytic activity and selectivity of a bimetallic catalyst may generally be explained based on the geometric and electronic effects and stability on stabilizing effects, while the improvement of reaction rates is related to the synergistic and bifunctional effects. Furthermore, in the presence of metal oxides, another effect due to the presence of the second metal is the introduction of a second functionality, such as acid or redox sites. This effect will depend on the degree of reduction of the metals present, considering that an incomplete reduction of metallic atoms can occur. Therefore, to improve the catalytic activity owing to the combination of electronic bifunctional effects, the development of supported bimetallic catalysts has been reported as a promising alternative to replacing one or both of the noble metals with less hazardous and lower-cost transition metals, such as Cu, Fe, Co, and Ni. Among these transition metals, much attention has been paid to the development of supported bimetallic catalysts based on Fe, Co, and Ni. However, despite its high potential in industrial applications, Cu has received less attention in the search for efficient catalysts for the electro-oxidation of glycerol (GlyEOR) mostly because of its tendency to form CuO resulting in low electronic conductivity and instability. Concerning the bimetallic Au–Cu catalyst, only a few studies have addressed its application for alcohol oxidation and even less for the GlyEOR, despite the wide use of individually supported gold and copper. Thia et al. (2016) reported the effect of the electrodeposition of Cu species onto Au/C on the enhancement of C3 selectivity of Au NPs during glycerol electro-oxidation; Redina et al. (2015) prepared Au–Cu bimetallic catalysts by a surface redox-reaction method in which Au NPs were directly deposited on the surface of copper oxide or Cu^2+^ species and tested in aerobic gas-phase ethanol oxidation. Wang et al. (2013) performed a systematic study on the oxidation of alcohols with molecular oxygen over supported gold catalysts, showing that Au/CuO prepared by a co-precipitation method is a highly active, selective, and stable heterogeneous catalyst. Sobczak and Wolski (2015) evaluated the effect of a copper species on the oxidation of glycerol in the presence of oxygen under pressure in a batch reactor using bimetallic Au–Cu on Nb_2_O_5_ and Nb/MCF supports, establishing an increase in the activity and selectivity of glycerol. Although many aspects of the catalytic activity of gold- and copper-based catalysts have been studied in the last 20 years, such as the particle size, morphology, and dispersion, the interaction between gold or copper and transition metal oxides, and methods of preparation, the role of the oxidation state of Au [mainly related to the instability of Au(III) and Au(I)] and Cu [mainly related to the instability of Cu(II) and Cu(I)] remains unclear to date (Minicò et al., 1997; Bond & Thompson, 2000; Wang et al., 2013a; Thia et al., 2016).

Another important strategy to improve the catalytic activity of bimetallic catalysts for the GlyEOR, with the aim to 1) increase conductivity, 2) reduce the electrical energy consumption by attaining the lowest possible potential domain, and 3) promote selective oxidation favoring desorption processes of GlyEOR products at the catalyst surface, has focused on the role of catalyst supports. The most studied supports are carbon black (C), activated carbon (AC), graphene (G), carbon nanotubes (CNTs), metal oxides, and polymers (Antolini, 2009). In spite of the fact that carbon black is being widely used as an electrocatalyst support, several drawbacks, such as low stability at temperatures above 100°C and the presence of a high amount of micropores, which can hinder the reactant flow, have led to the search for novel carbon nanomaterials. In recent years, the use of CNTs has dramatically increased because of their high crystallinity that confers to these materials the possibility of an excellent catalytic performance at high temperatures (>300°C), combined with a high surface area and amount of mesopores that aid in high metal dispersion (Novikova et al., 2018). To increase the efficiency of CNTs as support, strategies based on the functionalization of the external surface area have been implemented. These strategies primarily consist of the introduction of oxygenated functionalities, mostly carboxylic, nitro, and sulfonic groups, in the walls of CNTs (Wang et al., 2010; Esquivel-Peña et al., 2019). The second most studied supports are metal oxides because of their ability to provide oxygenated species to oxidize adsorbed intermediates. Fluorite-type metallic oxides, such as ceria, thoria, and zirconia, which present a face-centered cubic (FCC) crystal structure, have been extensively researched as conductive materials (Zhang et al., 2016). Liu and Flytsani-Stephanopoulos (1995) reported that, in the FCC crystal structure, each tetravalent oxide ion is surrounded by eight equivalent O^2–^ ions, and oxygen vacancies are created when a fluorite oxide is doped with divalent or trivalent ions. Consequently, the high oxygen vacancy concentration and related mobility properties have been proven to enhance catalytic activity. In the case of ceria, Ta et al. (2013) established that its unique properties arise from the fast and reversible Ce^4+^/Ce^3+^ redox cycle occurring in the fluorite lattice, where oxygen in the gas phase is transferred to the solid surface where the chemical reaction occurs. Furthermore, at the nanoscale, Mai et al. (2005) reported that oxygen storage and release can occur both at the surface and in the bulk of ceria nanorods and nanocubes, but only on the surface in the case of nanopolyhedral ceria. Several authors (Han et al., 2009; Tana et al., 2009), both from theoretical and experimental studies, have proposed that the superior oxygen storage capacity is linked to the fact that {100}- and {110}-dominated surfaces present in CeO_2_ nanorods are inherently more reactive than {111}-dominated surfaces present in polyhedral CeO_2_ NPs. Furthermore, when used as a support, the morphology of CeO_2_ NPs has been established to control the dispersion of metal particles and mediate metal-support interactions (Si & Flytzani-Stephanopoulos, 2008; Han et al., 2009; Boucher et al., 2011; Lin et al., 2011). In heterogeneous catalysis, the ability of the surface of an oxide support to interactions with oxygen, known as surface oxophilicity, has been found to be a very important variable in several catalytic and electrocatalytic reactions. The complete electrochemical oxidation of small organic molecules, such as glycerol on noble metal electrodes, requires an additional source of oxygen, provided by the catalytic surface, due to the adsorption of intermediate products. Several authors have indicated that more active catalysts can be obtained by properly controlling the coverage of the catalyst with oxygenated species (Yoo et al., 2010; Shan et al., 2013; Santos et al., 2021). In summary, CeO_2_ was identified as a reducible oxide owing to its facile redox properties and rich oxygen vacancies as well as other metal oxides that present the same characteristics, such as TiO_2_, Fe_2_O_3_, NiO, and MnO_2_. In the case of TiO_2_ as a support in the catalyst Au–TiO_2_, several authors, through theoretical and experimental studies, have concluded that surface hydroxyl groups on TiO_2_ promote O_2_ adsorption close to Au NPs, where they can react. This is a critical step for obtaining a structurally stable Au NP catalyst with substantial catalytic activity in the CO oxidation reaction (Liu et al., 2006; Veith et al., 2009). Moreover, studies on the adsorption and dissociation of H_2_O on TiO_2_ have concluded that water molecules on oxide surfaces are adsorbed on metal ions with the transfer of one of the protons to a neighboring oxygen atom, and OH groups mediate the interaction between the oxide surface and its surroundings (Kurtz et al., 1989; Hugenschmidt et al., 1994).

In view of the growing interest in supported bimetallic Au-based catalysts for the enhancement of catalytic activity in the GlyEOR as economical and eco-friendly materials, the present paper deals with the synthesis of Cu, Au, and Cu–Au NPs in previously functionalized multi-walled carbon nanotubes (MWCNTs). The process was performed by intermatrix synthesis for the charge of the first metal (Cu or Au) and galvanic replacement of Cu for the deposition of a second metal (Au) to form bimetallic (Cu–Au) NPs and further deposition in two metal oxides, CeO_2_ NPs and TiO_2_ NPs, to evaluate the interrelation between the preparation, controlled structure, and catalytic properties for their application in the GlyEOR in an alkaline medium. To the best of our knowledge, the role of key parameters, such as Cu and Au oxidation states, linked to the effects of the size, morphology, and metal composition of facile reducible oxides as supports (i.e., CeO_2_ and TiO_2_) that allow a better understanding of the properties of the obtained catalysts in the GlyEOR in alkaline media have not been well identified. A step-by-step characterization of the different stages of the synthesis and the final electrochemical evaluation of the catalytic activity via cyclic voltammetry (CV) were performed with the aim that the results contribute to providing a clearer insight into the achieved performance.

## 2 Materials and methods

### 2.1 Materials

All chemicals were of the highest grade available and were used as received without further purification. Raw MWCNTs were provided by SES Research (carbon purity >95%; OD 10–30 nm; length 5–15 μm). Metal nanoparticles were synthesized using inorganic salts AuCl_3_•3H_2_O (Sigma-Aldrich, >99.9%) and Cu(NO_3_)_2_•2.5H_2_O (Fermont, 95%). For metal oxide supports, ceria nanorods and nanobars were synthesized using Ce(NO_3_)_3_•6H2O, NaOH (J.T.Baker, 98%), HNO_3_ (J.T.Baker, 65%), and 2-propanol (J.T.Baker, 99.5%) or ethanol (J.T.Baker, 99.3%), for ceria nanorods urea [CO(NH_2_)_2_, AR grade] was also employed. For titania nanoparticles, titanium (IV) isopropoxide (Sigma-Aldrich, 97%) and EtOH were used. Other reagents were HCl (Sigma-Aldrich, 37%), H_2_O_2_ (J.T.Baker, 30%), and NaBH_4_ (Sigma-Aldrich, 98%). Dissolutions were prepared with deionized water from a Milli-Q system (resistivity 18.2 MΩ cm).

### 2.2 Catalyst preparation

#### 2.2.1 Functionalization of MWCNTs

MWCNTs were functionalized according to the procedure described in a previous work (Esquivel-Peña et al., 2019). Briefly, approximately 1.00 g of MWCNTs was weighed and transferred to a volumetric flask, where 60 mL of a mixture of 10:9 volume ratio of H_2_SO_4_:HNO_3_ was added while being kept under sonication, after which the acidic mixture was heated at 60°C for 90 min under constant magnetic agitation. Then, 1,200 mL of deionized water was added to stop the reaction, and the resulting suspension was filtered. The solid was washed with deionized water until a neutral pH was reached, dried under vacuum at 60°C for 24 h, and characterized by Fourier-transform infrared spectroscopy (FTIR).

#### 2.2.2 Synthesis of MNPs in MWCNTs

To prepare monometallic NPs, functionalized MWCNTs (50 mg) were immersed in 20 mL of 0.1 mol L^−1^ NaBH_4_, and the mixture was sonicated for 5 min. Subsequently, a known volume of the respective metal salt was added dropwise under constant sonication for 6 min. For Cu and Au NPs, 10 mL of a 20 mmol L^−1^ Cu(NO_3_)_2_ and 2 mL of 4.90 mmol L^−1^ HAuCl_4_•3H_2_O were used, respectively. At the end of the addition, sonication was performed for an additional 60 min. The remaining volume of NaBH_4_ (5 mL) was added dropwise under constant sonication, followed by sonication for an additional 60 min. The mixture was left to rest for 48 h and then filtered under vacuum. Finally, MNPs/MWCNTs were dried at 60°C for 24 h and characterized using FTIR, X-ray diffraction (XRD), and transmission electron microscopy (TEM). According to this procedure, the nominal composition of the Cu/MWCNT material was 20.4 wt% and that of the Au/MWCNT material was 3.0 wt%, assuming a total reduction of the metal precursors. The effective metal concentrations in the catalysts were determined by FAAS according to the procedure reported in Section 2.3.1.1. The nomenclature used consists of the chemical species followed by the nominal weight % in parenthesis to identify the electrocatalysts.

#### 2.2.3 Synthesis of bimetallic NPs (Cu–Au) in the MWCNT

These materials were obtained by the galvanic displacement of Cu by Au. Approximately 25 mg of the Cu/MWCNT material was obtained as previously described and was placed in a centrifuge tube, and 10 mL of deionized water was added to it. The mixture was dispersed, and the suspension was agitated for 5 min. Then, 1.0 mL of a 5.0 mM HAuCl_4_ was added dropwise under sonication. At the end of the addition, the suspension was sonicated for an additional 30 min, was allowed to rest for 24 h, and then vacuum filtered. Finally, bimetallic/MWCNTs were dried at 60°C for 48 h, and the selected samples were characterized by FTIR and TEM. According to the procedure, and assuming a 15% displacement of Cu by Au, the nominal compositions of Cu and Au were 18 and 3.0 wt%, respectively. The same procedure was used to obtain Cu–Au/MWCNT catalysts with varying amounts of Cu by modifying the initial volumes of the Cu(NO_3_)_2_ solution. The effective metal concentrations in the catalysts were determined by FAAS, according to the procedure reported in Section 2.3.1.1. The nomenclature used consists of the chemical species followed by the nominal weight % in parenthesis to identify the electrocatalysts.

#### 2.2.4 Synthesis of CeO_2_ NPs

First, the synthesis of CeO_2_ NPs was performed based on the hydrothermal method reported by Chen et al. (2008). In a typical preparation, 1.04 g of Ce(NO_3_)_3_ 6H_2_O and 2.16 g of urea were added to 480 mL of deionized water in a 1-L round-bottom flask under vigorous magnetic stirring. The solution was placed in a heating bath under reflux at 80°C and mechanically stirred at 300 rpm for 24 h. Subsequently, the solution was cooled to room temperature (RT). The obtained samples were centrifuged, washed with deionized water, and vacuum dried at 65°C for 24 h. Once dried, the precursor was ground using an agate mortar and pestle. The Ce(OH)CO_3_ nanoparticles obtained were re-dispersed into 120 mL of deionized water in a 1-L round-bottom flask under the same stirring conditions as previously described. After the addition of 14.4 g of NaOH, the solution was mechanically stirred at 300 rpm for 30 min at RT. After 4 days of aging, the solid was washed thrice with 25 mL of 1 mol L^−1^ HNO_3_, thrice with 25 mL of deionized water, and twice with 25 mL of ethanol and centrifuged at 3,000–5,000 rpm after each wash. Finally, the product was vacuum dried at 60°C for 24 h and ground using an agate mortar. The phase and morphology of the obtained solid were characterized using powder XRD and TEM.

The second approach reported by Phuruangrat et al. (2017), which involves obtaining CeO_2_ NPs by a microwave-assisted hydrothermal procedure, was used with some modifications. Approximately 1.08 g of Ce(NO_3_)_3_·6H_2_O was weighed and dissolved in 50 mL of deionized water. Thereafter, 3.0 mL of 3 mol L^−1^ NaOH solution and 7.0 mL of deionized water were added dropwise under stirring (approximately 11–13 min). Immediately, a precipitate was observed, which slowly turned to a purplish color, indicating the presence of Ce(OH)_3_(s). The solution was then agitated for 30 min. After that, 30 mL of the resultant solution was transferred to 60-mL Teflon vessels, which were sealed and placed in a microwave digester (CEM, MARS 6) running a heating program at 160°C for 80 min (20 min of ramp time to reach this temperature and 60 min of holding time at this temperature). After air-cooling to RT, the obtained solid was placed in 50-mL Falcon tubes and washed twice with 30 mL of 1 mol L^−1^ HNO_3_, 30 mL of deionized water, and ethanol. The tubes were vigorously shaken between each wash. The solid was recovered by centrifugation (VELAB prime centrifuge) at 5,000 rpm for 5 min. Finally, the product was vacuum dried at 70°C for 24 h. The phase and morphology of the obtained solid were characterized by powder XRD and TEM.

#### 2.2.5 Synthesis of TiO_2_ NPs

The procedure proposed by Bregadiolli et al. (2017), based on a hydrothermal microwave-assisted approach under alkaline conditions, was used with several modifications. In a 100-mL beaker containing 28 mL of 1 M NaOH, 12 mL titanium (IV) isopropoxide was added dropwise under magnetic stirring. After the addition was complete, the solution was stirred for 3 h at 70°C. Then, 20 mL of it was transferred to two 60-mL Teflon vessels, which were sealed and placed in a microwave digester (CEM, MARS 6) under a heating program at 160°C for 80 min (20 min of ramp time to reach this temperature and 60 min of holding time at this temperature). After air-cooling to RT for 15 min, the solutions obtained were placed in 50-mL Falcon tubes, centrifuged at 5,000 rpm for 5 min, and the solid was recovered by decantation. Subsequently, 20 mL of 1 mol L^−1^ HNO_3_ was added, and the samples were centrifuged at 5,000 rpm for 5 min and decanted. This procedure was repeated twice. Washing was performed with 20 mL of deionized water, followed by centrifugation (5,000 rpm for 8 min) and decantation, which was repeated until a pH of 5 was obtained. Subsequently, 20 mL of ethanol was added to the solid, followed by centrifugation (5,000 rpm for 5 min) and decantation. Finally, the product was vacuum dried at 70°C for 24 h. The obtained solid was characterized using powder XRD. However, at this point, profile fitting could not be performed because of the low crystallinity of the sample. As thermal treatment affects the dimensions, structure, and morphology of titanium nanostructures, the samples were subjected to further calcination at temperatures of 400, 600, and 800°C (Zavala et al., 2017). In this case, it was possible to observe a well-defined crystal structure from diffractograms.

#### 2.2.6 Synthesis of hybrid catalysts of MNPs/MWCNT–CeO_2_ (TiO_2_)

For the incorporation of the metallic oxide as a support to the Cu–Au/MWCNT catalyst, a “one pot” procedure was followed. For this procedure, approximately 25 mg of functionalized MWCNTs and 25 mg of CeO_2_ NPs were weighed and ground using an agate mortar to obtain the hybrid catalyst containing 50 wt% of CeO_2_. The solid obtained was placed in 50-mL Falcon tubes with 20 mL of 0.1 M NaBH_4_ and sonicated for 5 min, after which 10 mL 0.02 M of Cu(NO_3_)_2_ was added dropwise under sonication. After 60 min of sonication, an additional 5 mL of a 0.1 M NaBH_4_ was added, and the suspension was sonicated for 60 min, allowed to rest for 24 h, and vacuum filtered. The obtained solid was washed with deionized water until a pH value of 5–6 was obtained. The product was vacuum dried at 65°C for 24 h. Next, to approximately 25 mg of the obtained solid, 1.0 mL of a 5.0 mM HAuCl_4_ solution (see Section 2.2.3) was added dropwise while being kept under sonication. At the end of the addition, the suspension was sonicated for an additional 30 min, allowed to rest for 24 h, and vacuum filtered. Finally, the products were dried at 60°C for 48 h and characterized by SEM, STEM, and X-ray photoelectronic spectroscopy (XPS). The same procedure was used to obtain the Cu–Au/MWCNT–CeO_2_ catalyst with 25 and 10 wt% of CeO_2_, respectively, by modifying the initial amounts of MWCNTs and CeO_2_ NPs. In addition, to prepare the Cu–Au/MWCNT–TiO_2_ hybrid catalyst containing 10, 25, and 50 wt% TiO_2_ NPs as the support, the same method was adopted. The nomenclature used consists of the chemical species followed by the nominal weight % in parenthesis; the nature and amount of MO_2_ are also indicated for identifying the electrocatalysts.

### 2.3 Catalyst characterization

#### 2.3.1 Elemental and structural characterization

##### 2.3.1.1 Flame atomic absorption spectroscopy

After wet chemical treatment, a PerkinElmer 3100 FAA spectrometer was used to determine Cu and Au contents in MWCNTs according to the conditions established by the manufacturer, using a standard addition method. For wet chemical treatment, samples of selected materials were first homogenized in an agate mortar. For Cu and Au quantification, 30 mg of the sample was weighed in duplicate and 10 mL of 10% (v/v) HNO_3_ or aqua regia was added. The suspension was agitated and heated at 80°C until dry. Thereafter, another 5 mL of 10% (v/v) HNO_3_ was added. The resultant was agitated and heated until approximately 95% of the original volume was reduced. Then, 10 mL of 2% (v/v) HNO_3_ was added, and the suspension was made up to a volume of 50 mL with 2% (v/v) of HNO_3_ and filtered. The reagent blanks were prepared simultaneously.

##### 2.3.1.2 FTIR

Pristine and functionalized MWCNTs were characterized by FTIR analyses. Infrared spectra were obtained with a Perkin Elmer Spectrum GX FTIR spectrometer, for which MWCNTs were mixed with KBr to form a pellet using a mechanic press. The spectrum was acquired in the transmission mode with 50 scans.

##### 2.3.1.3 X-ray diffraction

XRD patterns were collected using a Bruker AXS D8 ADVANCE DAVINCI diffractometer with a Cu K*α* X-ray source in a Bragg–Brentano configuration. The as-prepared catalysts were analyzed by XRD without further modification.

##### 2.3.1.4 Electron microscopy

The images were obtained using a JEOL JEM-2010 transmission electron microscope with a LaB_6_ thermionic gun at 200 kV. Image analysis was performed with the ImageJ software using background subtraction, bandpass filters, and manual color adjustment to increase the MNP brightness with respect to the carbon support. STEM images were obtained using a JEOL JSM-6510LV scanning electron microscope with a LaB_6_ thermionic gun in the low voltage mode (5 kV). EDS spectra and elemental mapping were recorded at 15 eV using the same equipment. For sample preparation, a 0.001 wt% suspension of each material in isopropyl alcohol was prepared by ultrasonication. Subsequently, 2.5 μL of the suspension were deposited on a 200-mesh nickel grid with a lacey carbon support structure and was dried at room temperature.

##### 2.3.1.5 X-ray photoelectronic spectroscopy

X-ray photoelectronic spectroscopy was performed using the ESCALAB 250Xi instrument equipped with an Al Kα X-ray source (1,486.68 eV). The pressure in the chamber was maintained at 10 bars during the measurements. A high-resolution spectral survey was conducted with the pass energy of 20 eV. Spectra deconvolution was performed using CasaXPS software, where C 1 s = 284.5 eV, set as the calibration value for binding energy. A combination of Lorentzian and Gaussian peak shapes was used for the deconvolution of signals.

#### 2.3.2 Electrochemical characterization

Electrochemical experiments were performed using a Methrom Autolab PGSTAT302N (Nova 1.11, 2004) at RT (23°C ± 1°C). A 10-cm^3^ glass cell with a three-electrode configuration was used. Hg/HgO (filled with a 1 M NaOH solution) and a platinum wire were used as the reference and counter electrodes, respectively. The working electrode was prepared by mixing 5 mg of the catalyst with 425 μL of deionized water, 50 μL of the Nafion® solution (in 5% aliphatic alcohol, Electrochem), and 50 μL of isopropanol in an ultrasonic bath for 20 min. From the resulting ink, 5 μL was deposited on a glassy carbon electrode, which was previously mirror-polished, with a cross-sectional area of 0.071 cm^2^ (*n* = 3). The electrodes were then dried overnight under ambient conditions. To determine the catalytic activity of Cu/MWCNT, Au/MWCNT, Cu–Au/MWCNT, and Cu–Au/MWCNT–MO_2_ for glycerol oxidation, CV was performed.

## 3 Results and discussion

### 3.1 Metal content in catalysts

The chemical composition of the prepared monometallic and bimetallic catalysts supported in previously functionalized MWCNTs, and in the organic–inorganic support composed of functionalized MWCNTs and CeO_2_ or TiO_2_, was determined by FAAS according to the procedure described in Section 2.3.1.1. In Table 1, the nominal and the effective concentrations of Au and Cu are given for the reported catalysts and Au/Cu atomic ratios, which vary from 0.032 to 0.113. The difference observed between the nominal concentration of Au (3 wt%) and the mean effective concentration in all the prepared catalysts allows us to conclude that the preparation procedure is reproducible and reliable. As for Cu, the experiments were designed with loadings varying from 10–30 wt%. However, uncertainty values are in the ± 1% (except for the catalyst with a loading of 30 wt%).

**TABLE 1:** Gold and copper nominal and effective loadings.

Catalyst^a^	Au, wt% nominal	Au, wt% FAAS	Cu, wt% nominal	Cu, wt% FAAS	Atomic ratio Au/Cu
Au(50)/MWCNT	50	52^a^ (TGA)	―	―	―
Au(6)/MWCNT	6	5.80 ± 0 .26	―	―	―
Au(3)/MWCNT	3	3.87 ± 0.06	―	―	―
Cu(20)/MWCNT	―	―	20	22.29 ± 0.91	―
Cu(28)Au(3)/MWCNT	3	3.63 ± 0.09	28	29.31 ± 2.02	0.040
Cu(18)Au(3)/MWCNT	3	3.64 ± 0.19	18	21.97 ± 0.81	0.053
Cu(9)Au(3)/MWCNT	3	3.36 ± 0.22	9.0	9.57 ± 0.35	0.113
Cu(18)Au(3)/MWCNT–CeO_2__rods (50%)	3	3.30 ± 0.18	18	20.50 ± 0.51	0.052
Cu(18)Au(3)/MWCNT–CeO_2__bars (10%)	3	3.05 ± 0.15	18	19.41 ± 0.74	0.051
Cu(18)Au(3)/MWCNT–CeO_2__bars (25%)	3	3.06 ± 0.08	18	18.33 ± 0.64	0.054
Cu(18)Au(3)/MWCNT–CeO_2__bars (50%)	3	3.28 ± 0.21	18	18.72 ± 0.55	0.056
Cu(28)Au(3)/MWCNT–TiO_2_ (10%)	3	3.45 ± 0.02	28	25.59 ± 1.0	0.043
Cu(28)Au(3)/MWCNT–TiO_2_ (25%)	3	2.55 ± 0.09	28	25.98 ± 0.80	0.032
Cu(18)Au(3)/MWCNT–TiO_2_ (25%)	3	2.95 ± 0.34	18	20.52 ± 0.82	0.046
Cu(28)Au(3)/MWCNT–TiO_2_ (50%)	3	3.17 ± 0.09	28	26.85 ± 0.52	0.038

^a^ For the identification of electrocatalysts, the nomenclature used consists of the chemical species followed by the nominal weight % in parenthesis.

### 3.2 Metal nanoparticle-carbon nanotubes

#### 3.2.1 Structural characterization of Cu/MWCNTs, Au/MWCNTs, and Cu–Au/MWCNTs

##### 3.2.1.1 FTIR

**FIGURE 1:**
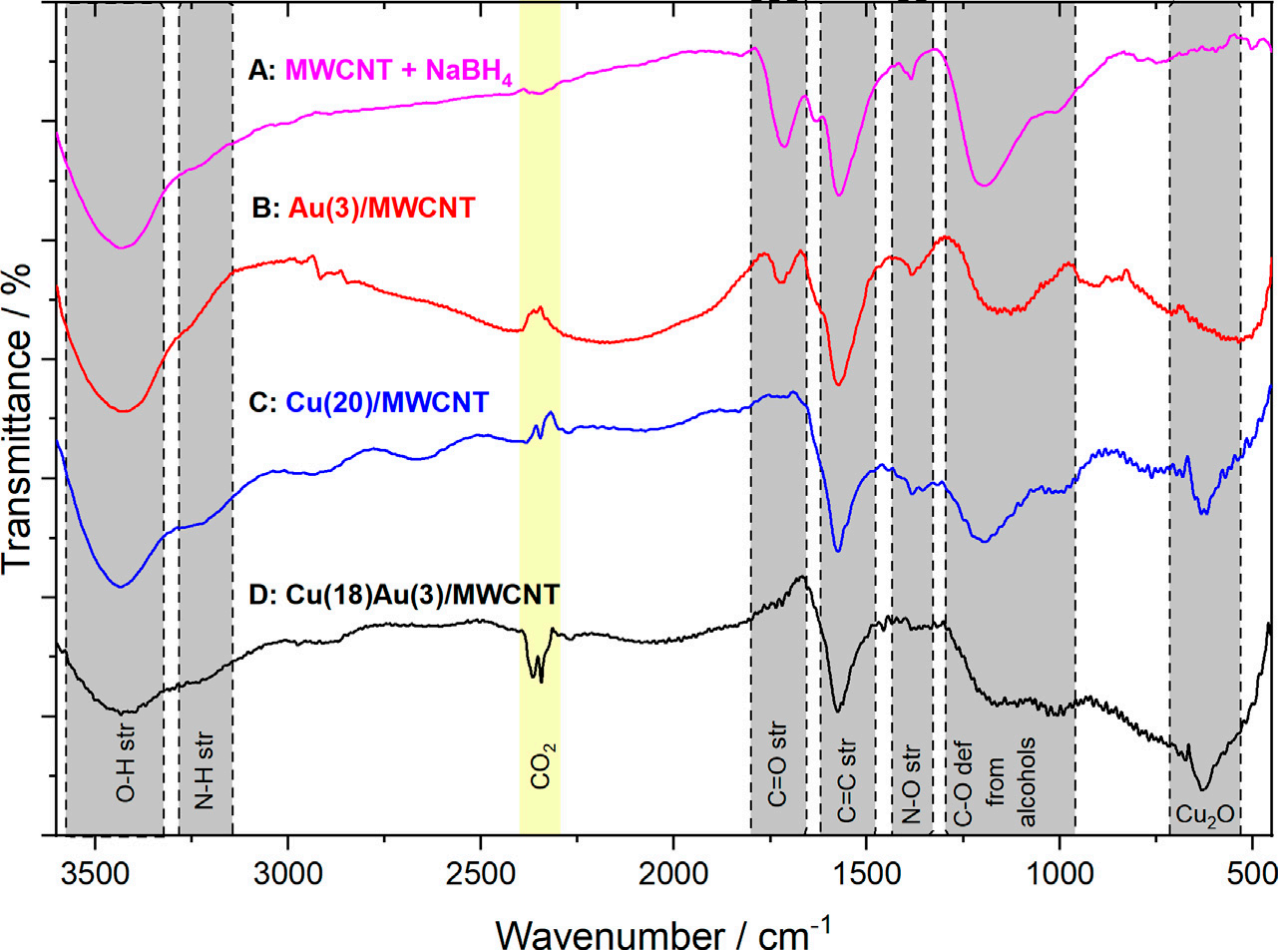
FTIR spectra for the obtained materials: **(A)** functionalized MWCNTs treated with NaBH_4_ (MWCNT + NaBH_4_), **(B)** MWCNT + NaBH_4_ in the presence of Au NPs (Au(3)/MWCNTs), **(C)** MWCNT + NaBH_4_ in the presence of Cu NPs (Cu(20)/MWCNTs), and **(D)** MWCNT + NaBH_4_ in the presence of Cu–Au NPs (Cu(18)Au(3)/MWCNT).

The MWCNTs were functionalized according to a previously reported procedure by Esquivel-Peña et al. (2019). Figure 1 shows the IR spectra of functionalized MWCNTs after the addition of NaBH_4_ and in the presence of Au NPs, Cu NPs, and Cu–Au NPs; the main band assignments are given in Supplementary Table S1.

From the spectrum of functionalized MWCNTs treated with NaBH_4_ (Figure 1A), a strong broad band in 3,480–3,430 cm^−1^ is assigned to O–H stretching and a shoulder at 3,280–3,220 cm^−1^ to N–H stretching. The signals at 1,725 cm^−1^, 1,575 cm^−1^, and 1380 cm^−1^ are assigned to C=O stretching of carboxylic acids, C=C stretching from the main structure of MWCNTs, and N–O stretching, respectively. The broad signal at 1,200–1,095 cm^−1^ is related to C–O stretching from alcohols. With the addition of Au [Au(3)/MWCNT, Figure 1B], the peak at 3,434 cm^−1^ is associated with the presence of OH/NH groups, with an evident decrease in the 3,220 cm^−1^ peak. The signal at 1,725 cm^−1^ assigned to C=O stretching of carboxylic acids (also present in MWCNTs + NaBH_4_) indicated that not all carboxylic acids were reduced to alcohols, probably due to the small amount of Au added. It is interesting to observe a shoulder at 1,630 cm^−1^ next to C=C stretching, which confirms the presence of Au NPs due to the interaction of carbonyl groups with metallic gold (Socrates, 2005). Additionally, the broadening of signals between 1,200–1,015 cm^−1^, together with the displacement of the signal initially at 681–674 cm^−1^, may be due to Au–OH interactions. After the incorporation of Cu NPs to MWCNTs + NaBH_4_ [Cu(20)/MWCNT], where Cu NPs are synthesized by adding Cu(NO_3_)_2_ and then NaBH_4_ (Figure 1C), the peak at 3,434 cm^−1^, such as in Au NP spectra, is assigned to the elongation of the O–H bond present in carboxylic acids and water of crystallization. The peak at 3,220 cm^−1^ is assigned to N–H stretching. The signals observed at 1,574 and 1,200–1,015 cm^−1^ are assigned to C=C stretching and C–O stretching from alcohols, respectively. The absence of the signal at 1725 cm^−1^ indicates the reduction of carboxylic acids to alcohols by NaBH_4_ catalyzed by the presence of Cu NPs. With the addition of HAuCl_4_ for the galvanic displacement of Cu to obtain the bimetallic catalyst [Cu(18)Au(3)/MWCNT], in the FTIR spectrum (Figure 1D), the decrease of signals at 3,480–3,150 cm^−1^ is observed together with the disappearance of the peaks at 1,382 and 1,196 cm^−1^, previously described in Figure 1C, indicating that the amine groups initially present were probably oxidized to alcohols by Au(III) according to the mechanism reported by Adenier et al. (2004) and Rahman et al. (2000). Furthermore, the interactions of Au NPs with MWCNTs are observed in Figure 1B. Finally, the peak corresponding to Cu_2_O (630 cm^−1^) remains in the spectrum. In summary, the FTIR results obtained clearly reveal the presence of Cu as Cu_2_O in the as-obtained catalysts. These observations are further discussed based on XRD and XPS results.

##### 3.2.1.2 XRD

**FIGURE 2:**
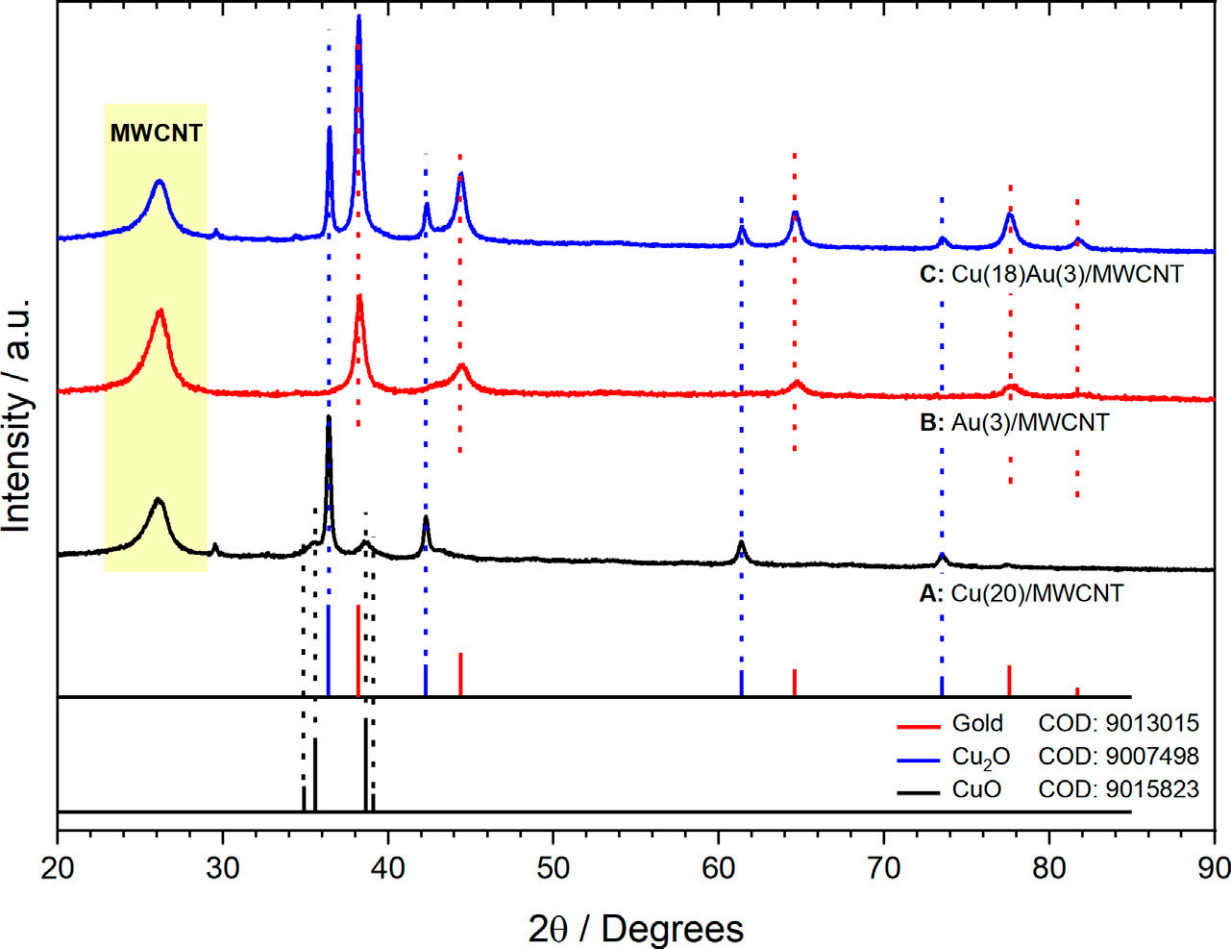
X-ray diffraction patterns for **(A)** Cu(20)/MWCNTs, **(B)** Au(3)/MWCNTs, and **(C)** Cu(18)Au(3)/MWCNTs.

X-ray diffraction patterns of mono- and bimetallic NPs supported on MWCNTs are shown in Figure 2, where good integration between the support and the metallic phase is observed, as previously reported in the study by Esquivel-Peña et al. (2019). As can be seen in Figure 2A, the Cu/MWCNT sample is a mixture of crystalline phases of metallic Cu, Cu_2_O, and CuO. However, the main component is Cu_2_O in an FCC crystal structure with a particle size of approximately 23 nm, as estimated by the Scherrer equation (Eq. 1), where τ is the average crystallite size, K is a constant with a value of 0.9, λ is the X-ray wavelength, β is the FWHM in radians, and θ is the diffraction angle. In addition, small-sized metallic Cu particles of approximately 6 nm are present alongside larger CuO particles (∼30 nm). The presence of CuO can be explained by the high tendency of metallic copper to oxidize and the disproportionation reaction of Cu(I). It is important to note that the Cu(I) oxidation state was stabilized by its interaction with MWCNTs, probably with the amine groups formed after the addition of NaBH_4_, as observed by FTIR. In Figure 2B, for the sample containing Au NPs, only metallic gold with a particle size of 10 nm is found to be above the detection limit of XRD.

$$\tau =\frac{K\lambda }{\beta \cos\theta }$$ (1)

As shown in Figure 2C, only Cu_2_O and Au(0) crystalline phases are present after galvanic displacement, which suggest that the gold phase covered Cu_2_O NPs, preventing their oxidation by the environment. Based on these results, the galvanic displacement reaction can be described using Eq. 2.

$$2AuCl_{4}^{-}+3Cu_{2}O+6H^{+}\to 2Au+6Cu^{2+}+{3H}_{2}O+8Cl^{-}$$ (2)

##### 3.2.1.3 Electron microscopy

**FIGURE 3:**
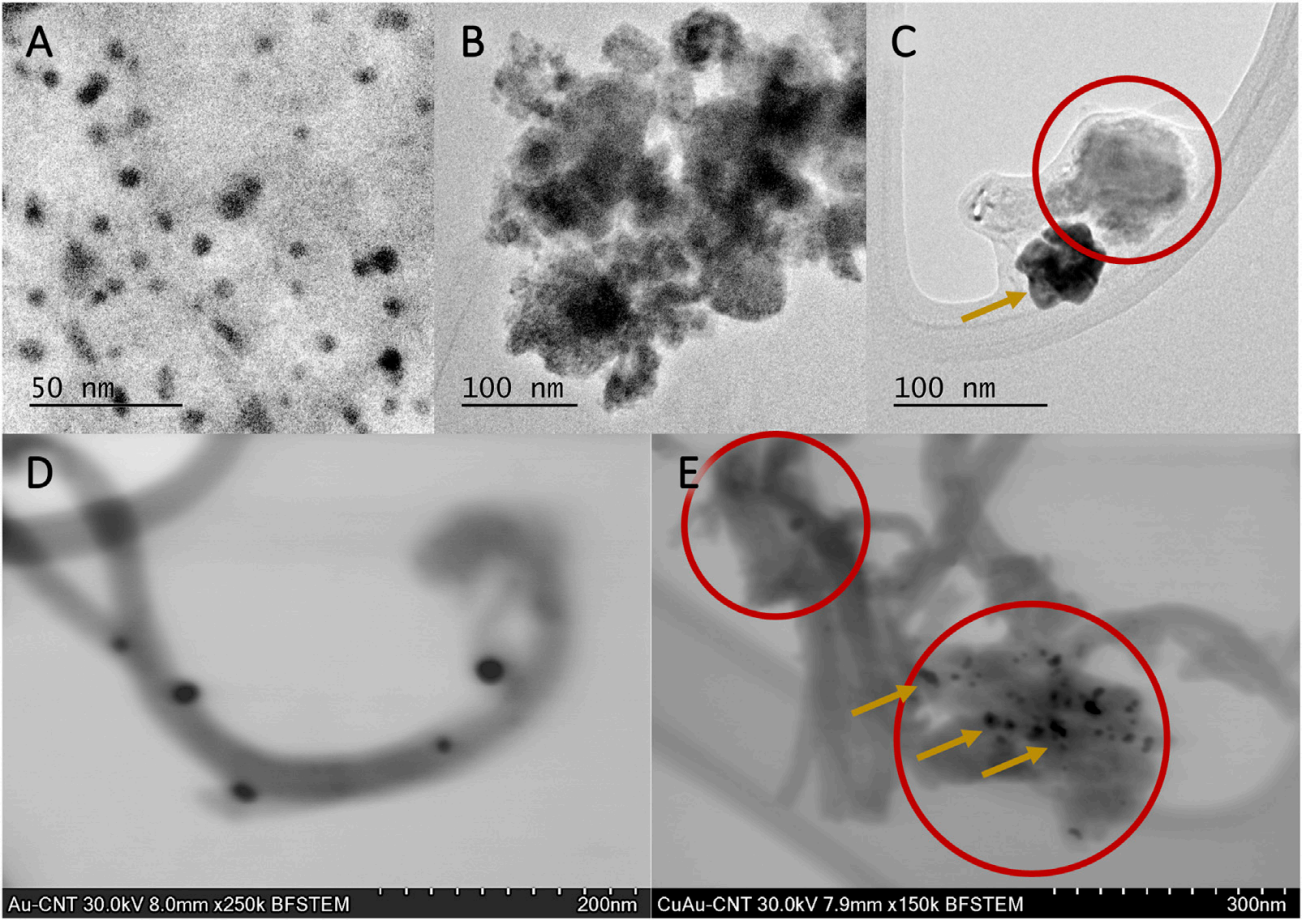
TEM images of **(A)** Au(3)/MWCNTs, **(B)** Cu(20)/MWCNTs, and **(C)** Cu(18)Au(3)/MWCNTs and STEM images of **(D)** Au(3)/MWCNTs and **(E)** Cu(18)Au(3)/MWCNTs. Cu NPs are indicated by circles and Au NPs by arrows.

TEM and STEM images of mono and bimetallic NPs supported on MWCNTs are shown in Figure 3. In Figures 3A,D, the structure of the Au/MWCNT catalyst is shown by TEM and STEM micrographs, respectively. Spherical particles of around 10 nm are observed homogenously distributed along the MWCNT matrix (Figure 3A); it is clearly observed that Au NPs (black dots) are supported on the surface of MWCNTs. In Figure 3B, small Cu_2_O NPs agglomerated to form larger sponge-like aggregates with a high specific area (encircled area), giving rise to an increase in roughness, which is a desirable characteristic for enhancing the catalytic activity of heterogeneous catalysts. The formation of Cu_2_O and, consequently, the stabilization of this morphology are promoted by the synthesis procedure of the Cu/MWCNT used in this work. Figures 3C,E correspond to TEM and STEM micrographs for the Cu(18)Au(3)/MWCNT catalyst, respectively. From TEM, in the case of bimetallic NPs, an apparent phase separation between Au and Cu NPs was observed. However, from STEM images, the coating of Au over Cu_2_O aggregates became evident, allowing the visualization of the increase in roughness.

#### 3.2.2 Electrochemical characterization of Cu/MWCNTs, Au/MWCNTs, and Cu–Au/MWCNTs

**FIGURE 4:**
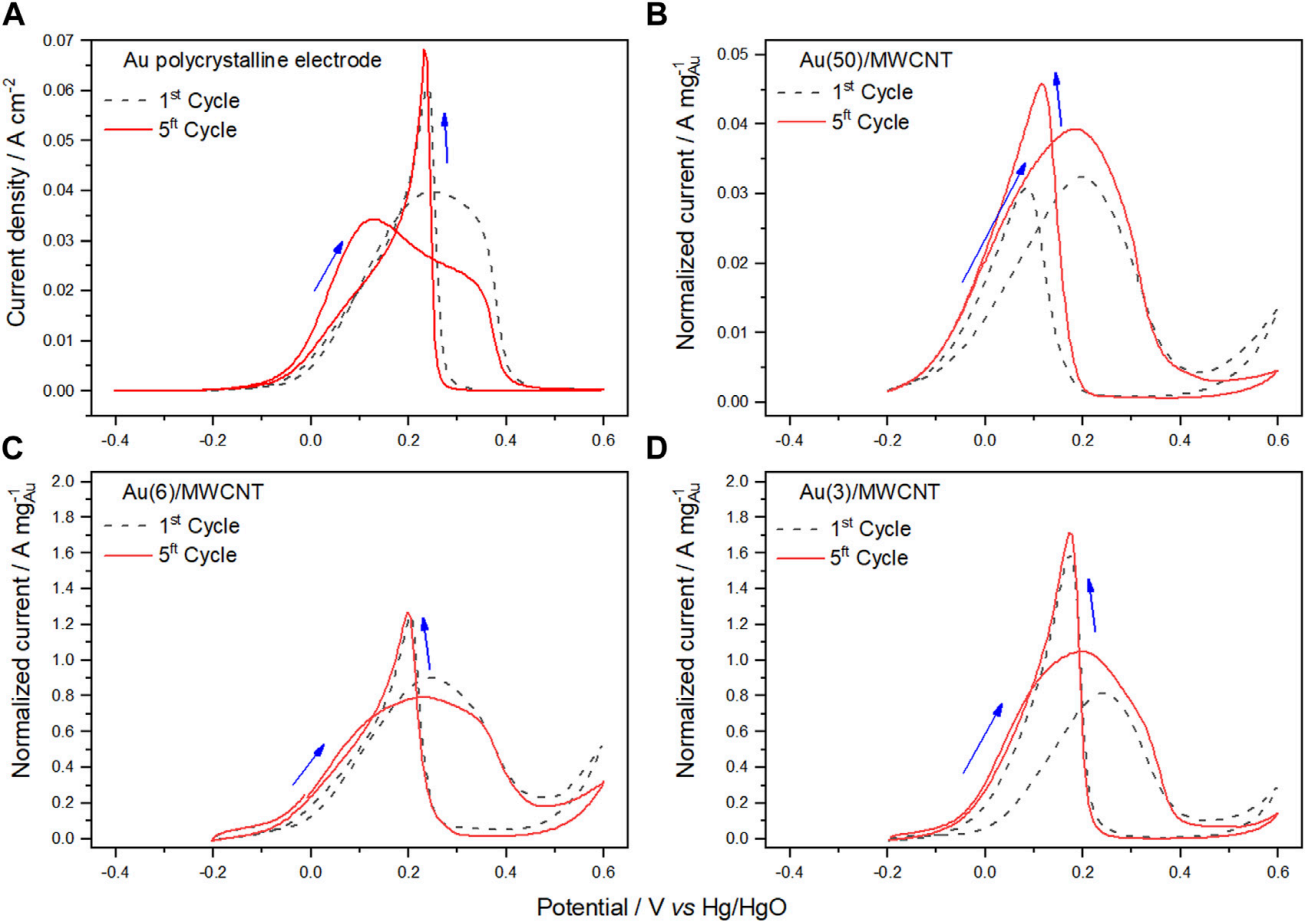
Cyclic voltammograms of Au electrocatalysts corresponding to the first and fifth cycle of glycerol oxidation in 0.5 mol L^−1^ NaOH solution containing 0.1 mol L^−1^ glycerol (sweep rate of 50 mV s^−1^ at 25°C) obtained for a polycrystalline Au electrode **(A)** and Au/MWCNTs with varying amounts of Au: **(B)** 50, **(C)** 6, and **(D)** 3 wt%.

Electrochemical characterization was performed using CV in the presence of glycerol in alkaline media. In Supplementary Figure S1, the cyclic voltammogram obtained for the Cu(20)/MWCNT catalyst in a 0.5 mol L^−1^ sodium hydroxide solution containing 0.1 mol L^−1^ glycerol is presented, where the absence of signals confirms the null electro-character of Cu in the GlyEOR. In contrast, Figures 4A–D display the cyclic voltammograms obtained for a polycrystalline Au electrode and Au/MWCNT electrocatalysts containing different amounts of Au NPs, under the same experimental conditions. From the voltammograms obtained for the polycrystalline Au electrode after the first and fifth cycle, as shown in Figure 4A, it is observed that, on the forward sweep, oxidation of glycerol begins at approximately −64 mV (forward onset potential, E_of_). The process continues until the formation of the gold oxide monolayer renders the gold surface inactive, and then, a decrease in glycerol oxidation occurs. In the case of the first cycle, a one-step relatively broad peak (E_pf_ 244 mV) was observed, which may be explained based on the most probable rate-determining step oxidation reaction of glycerol given by Eq. 3 (Simões et al., 2012; Srejić et al., 2016).

$$CH_{2}OH–CHOH–CH_{2}OH+5\;OH^{-}\;⇄\;CH_{2}OH–CHOH–COO^{-}+4\;H_{2}O+4\;e^{-}$$ (3)

Furthermore, according to Eq. 3 and considering that OH^−^ adsorption/desorption occurs as a reversible process in the same potential region, a second oxidation reaction represented by Eq. 4 explains the definition of two signals after the fifth sweep:

$$CH_{2}OH–CHOH–CH_{2}OH+2\;OH^{-}\;⇄\;CH_{2}OH–CHOH–CH=O+2\;H_{2}O+2\;e^{-}$$ (4)

However, this behavior may arise owing to interfacial phenomena in the proximity of the catalyst because of other reactive species. Adžić & Avramov-Ivić, (1986) proposed that the rate-determining step of Au in the GlyEOR in alkaline media is the adsorption of hydroxyl ions with partial electron transfer, which is sensitive to the different crystallographic facets of the Au surface. For the reverse sweep, the reduction of gold oxide begins at approximately 320 and 287 mV (E_ob_) for the first and last (fifth) cycle, respectively, allowing the surface to reactivate for further oxidation of glycerol and other reactive species. This results in another oxidation peak, which is practically present at the same oxidation potential (232 mV) and with the same peak current (0.068 mA cm^−2^), which is independent of the number of scans.

For the Au/MWCNT catalyst containing varying amounts of Au NPs (Figures 4B–D), the voltammograms for the first cycle of the GlyEOR show that E_onset_ on the forward sweep is displaced from −135 to −38 mV, while E_pf_ is displaced from 198 to 242 mV as the amount of Au decreases. Concerning the fifth cycle, a profile similar to that of the forward sweep in the first cycle of the Au polycrystalline electrode is observed. However, in the reverse sweep, the oxidation peak (E_pb_) appears at lower potentials (Table 2). Noticeably, as the amount of Au diminishes, the normalized peak current (J_p_) increases, obtaining the highest value for the catalyst with 3 wt%. This effect can be attributed to the decrease of the particle size as determined by XRD (90, 10, and 10 nm for Au(50)/MWCNTs, Au(6)/MWCNTs, Au(3)/MWCNTs, respectively), since small particle sizes result in a high specific area.

**TABLE 2:** Electrochemical parameters for the first and fifth cycle for mono- and bimetallic catalysts.

Catalyst	First cycle						Fifth cycle					
	Forward scan			Backward scan			Forward scan			Backward scan		
	E_onset_ (mV)	E_pf_ (mV)	J_pf_ (A mg^−1^_Au_)	E_onset_ (mV)	E_pb_ (mV)	J_pb_ (A mg^−1^_Au_)	E_onset_ (mV)	E_pf_ (mV)	J_pf_ (A mg^−1^_Au_)	E_onset_ (mV)	E_pb_ (mV)	J_pb_ (A mg^−1^_Au_)
Au polycrystalline	−64	244	0.040^a^	320	240	0.062^a^	−93	129	0.034^a^	287	232	0.068^a^
Au(50)/MWCNT	−135	198	0.032	247	85.5	0.031	−145	186	0.039	232	117	0.046
Au(6)/MWCNT	−95	232	0.776	317	181	1.299	−147	183	0.677	272	171	1.154
Au(3)/MWCNT	−38	242	0.815	313	172	1.594	−103	197	1.047	270	172	1.717
Cu(28)Au(3)/MWCNT	−14	283	0.954	233	152	0.317	−24	273	1.137	247	147	0.412
Cu(18)Au(3)/MWCNT	ND	244	0.265	340	149	0.284	−48	256	0.556	319	132	0.292
Cu(9)Au(3)/MWCNT	48	241	0.031	313	135	0.090	−45	215	0.169	310	110	0.123

^a^ In the case of Au polycrystalline electrodes, current peaks are given in mA cm^−2^ considering the electroactive area.

**FIGURE 5:**
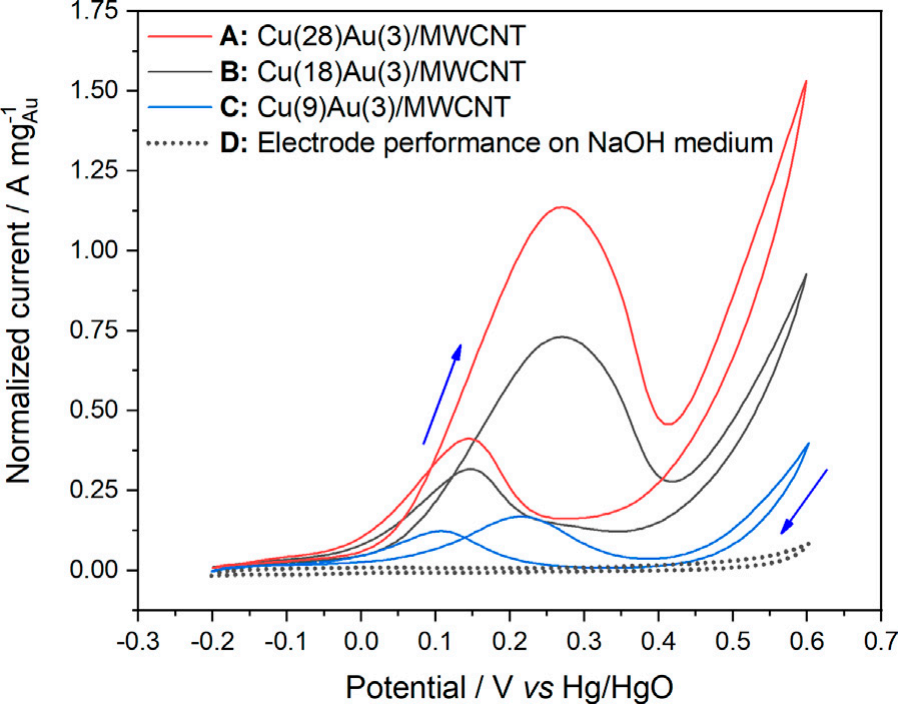
Cyclic voltammograms of bimetallic catalysts: **(A)** Cu(28)Au(3)/MWCNTs, **(B)** Cu(18)Au(3)/MWCNTs, and **(C)** Cu(9)Au(3)/MWCNTs in 0.5 mol L^−1^ NaOH solution containing 0.1 mol L^−1^ glycerol (sweep rate of 50 mV s^−1^ at 25°C) and **(D)** Cu–Au/MWCNTs in 0.5 mol L^−1^ NaOH solution.

In the case of bimetallic catalysts with 3 wt% of Au (Figure 5) and varying amounts of Cu, Cu(9)Au(3)/MWCNTs, Cu(18)Au(3)/MWCNTs, and Cu(28)Au(3)/MWCNTs, the voltammograms for each of the fifth cycle presents a very similar profile, with only an increase in the normalized peak current as the amount of Cu increases (J_pf_ values of 0.169, 0.556, and 1.137 A mg^−1^, respectively). The normalized peak current for the Cu(28)Au(3)/MWCNT catalyst is also higher than that for the Au(3)/MWCNT catalyst (1.047 A mg^−1^), indicating that more molecules of glycerol are being oxidized per unit mass of gold. Conversely, the average oxidation potential for Cu(9,18,28)–Au(3)/MWCNT catalysts in the forward sweep (248 ± 30 mV) is higher than that for Au(3)/MWCNT catalysts (197 mV), probably owing to the electrical insulation properties of Cu_2_O that retard the GlyEOR.

According to several authors (Marshall et al., 2015; Chung et al., 2016), the activity toward the electro-oxidation of alcohols is related to the ratio of the current density of forward and backward sweeps (J_pf_/J_pb_), which critically depends on the oxophilicity of the surface. Lai et al. (2021) recently proposed that the ratio of the integrated current of the forward oxidation peak to the integrated current of the backward peak can be interpreted as the reducibility of the oxidized metal surface formed at high potentials. Based on the values of the integrated current of forward and backward peaks (A_pf_ and A_pb_) obtained from the voltammograms of Au(3)/MWCNT and Cu(9,18,28)–Au(3)/MWCNT catalysts (Table 2), the corresponding A_pf_/A_pb_ ratios are listed in Table 3).

**TABLE 3:** Integrated current peak ratios for the first and fifth cycle of monometallic Au/MWCNT catalysts with varying amounts of Au and bimetallic Au–Cu/MWCNT catalysts with constant Au and varying amounts of Cu.

Catalyst	A_pf_/A_pb_ first cycle	A_pf_/A_pb_ fifth cycle
Au(3)/MWCNT	0.85	1.42
Au(6)/MWCNT	0.96	1.42
Au(50)/MWCNT	1.34	1.65
Cu(9)Au(3)/MWCNT	0.11	1.54
Cu(18)Au(3)/MWCNT	0.60	2.37
Cu(28)Au(3)/MWCNT	4.47	4.16

As observed for the monometallic Au/MWCNT catalyst, as the amount of Au increased, the A_pf_/A_pb_ ratio increased in the first and fifth cycle. In the case of the bimetallic catalyst, an important contribution of Cu is evidenced by the steep increase observed of this parameter in both cycles. With a variation of 3× the amount of Cu, the A_pf_/A_pb_ ratio almost triplicates its value, demonstrating the benefit of adding Cu as a second metal to the electrocatalyst composition. The increase in the ratio can be explained by the higher oxophilicity of Cu over Au (0.2 and 0.0, respectively) (Kepp, 2016), together with the presence of Cu NPs as Cu_2_O with a mesoporous structure (Figures 3A, B). The mesoporous structure was most probably derived from the particular conditions of the preparation method employed. Thus, a combination of factors led to the enhancement of the electrochemical active surface area, and consequently, a higher number of catalytic sites were available in the catalyst to facilitate the number of oxidized molecules of glycerol.

### 3.3 Metal nanoparticle–carbon nanotube–reducible oxide

#### 3.3.1 Reducible oxide

##### 3.3.1.1 Structural characterization

###### 3.3.1.1.1 CeO_2_

**FIGURE 6:**
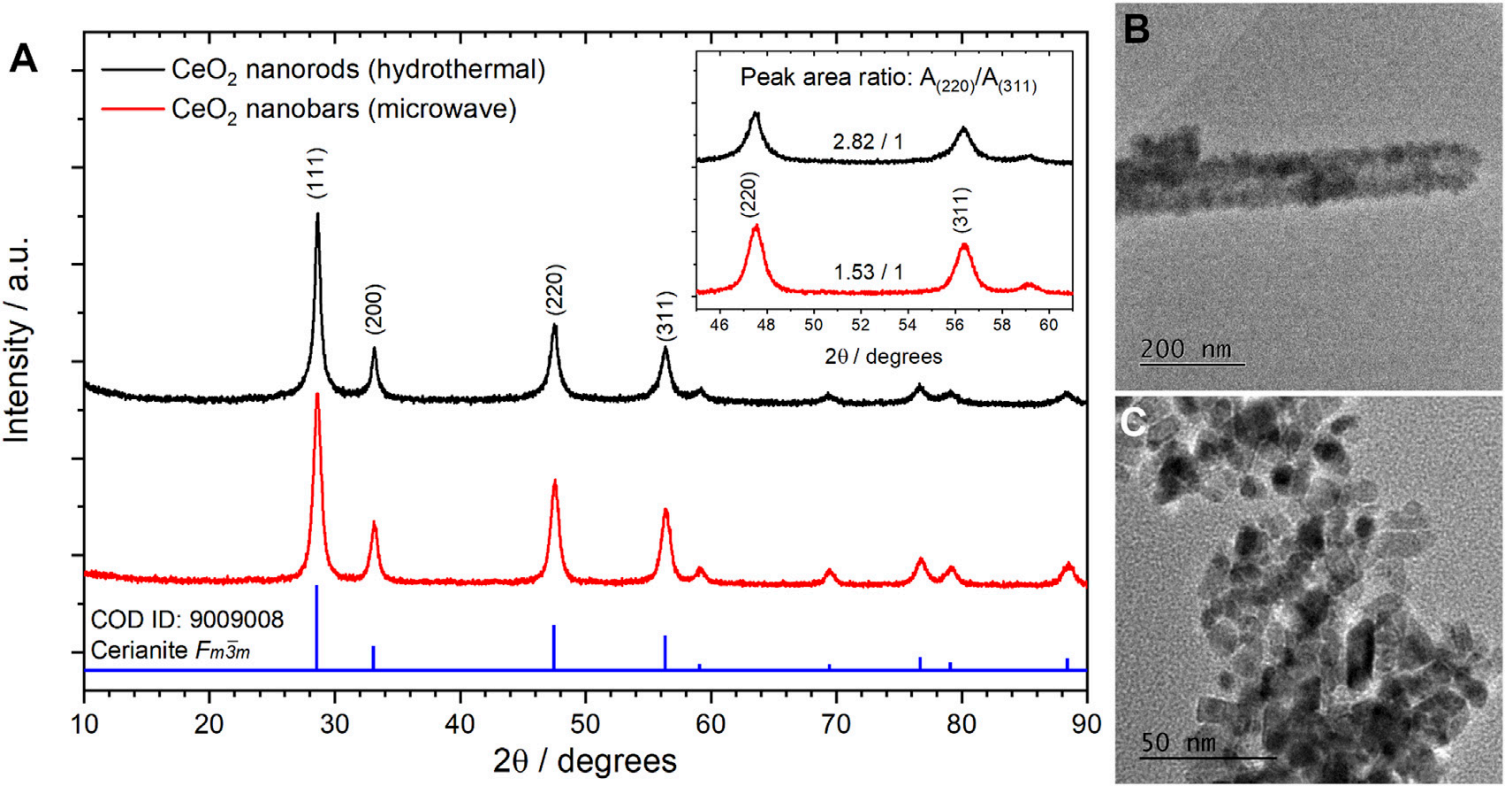
X-ray diffractograms **(A)** and TEM images of CeO_2_ nanoparticles obtained by non-surfactant hydrothermal **(B)** and microwave-assisted **(C)** procedures.

In Figure 6A, the X-ray diffraction patterns of CeO_2_ NPs are shown. It can be observed that both procedures used (Section 2.2.4.) lead to a cerianite crystal structure. The mean crystallite size obtained by the Scherrer equation using the (111) peek was found to be 9.6 and 9.2 nm for the non-surfactant hydrothermal and hydrothermal microwave-assisted methodologies, respectively.

CeO_2_ NPs tend to grow in the direction of [110], increasing the ratio between the integrated area for (220) and (311) peaks, [A_(220)_/A_(311)_]. In the case of the non-surfactant procedure, this type of growth indicates the presence of elongated NPs in a rod-like nanostructure; while in the case of the microwave-assisted procedure, a lower value indicates the presence of nanobars. In general, it has been observed that more elongated nanostructures such as rods perform better in the GlyEOR. Despite the results obtained, it is worth mentioning that the microwave-assisted procedure is more eco-friendly, less time-consuming, and allows for a higher yield than the non-surfactant procedure. Thus, in the current study, further experiments were performed with nanobars obtained using the microwave-assisted procedure. TEM images of synthesized nanomaterials were obtained to determine their size and morphology. Figure 6B reveals the presence of CeO_2_ nanorods with a mean diameter of 92 ± 15 nm and uniform lengths ranging from 750 to 2,200 nm. Conversely, in Figure 6C, CeO_2_ NPs present a bar-like shape with an average width and length of 13.5 ± 2.1 nm and 7.3 ± 1.9 nm, respectively. This confirmed the conclusions obtained from the XRD data.

###### 3.3.1.1.2 TiO_2_

**FIGURE 7:**
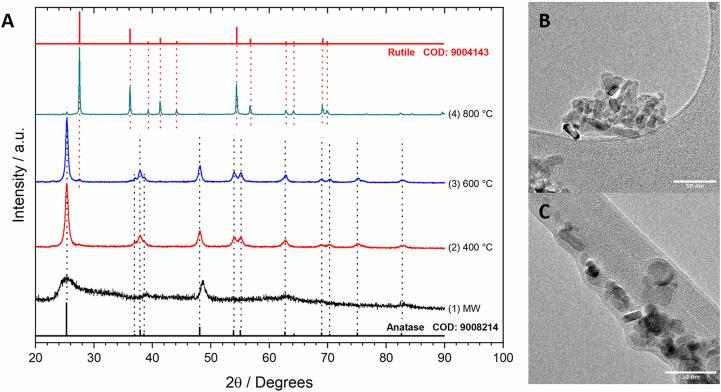
XRD patterns of TiO_2_ NPs obtained by hydrothermal microwave-assisted procedures **(A)** with no thermal treatment (1) and calcination at 400°C (2), 600°C (3), and 800°C (4), and TEM images of TiO_2_ NPs obtained after calcination at 400°C **(B,C)**.

Hydrothermal synthesis of TiO_2_ NPs usually results in amorphous materials; thus, calcination is recommended. For the synthesis of TiO_2_ NPs, a microwave-assisted hydrothermal procedure was followed as described in Section 2.2.5., and the XRD results are presented in Figure 7.

The XRD pattern of TiO_2_ NPs obtained without calcination (no thermal treatment) showed broad signals corresponding to anatase and other amorphous phases remaining even after thorough washing. However, crystalline phases were observed after calcination. At 400°C, only the anatase phase was present. At higher temperatures, the conversion to rutile took place, and rutile became the main component at 800°C. Several authors have reported anatase as the most active crystalline phase of TiO_2_ in the GlyEOR. TEM images of nanorods with a mean diameter of 12.3 ± 5 nm and uniform lengths ranging from 15.4 to 61.0 nm were observed. Derived from these results, in further works, TiO_2_ obtained by calcination at 400°C was used as support for Cu–Au/MWCNT catalysts.

#### 3.3.2 Cu–Au/MWCNT/CeO_2_ and Cu–Au/MWCNT/TiO_2_ catalysts

##### 3.3.2.1 Electron microscopy

**FIGURE 8:**
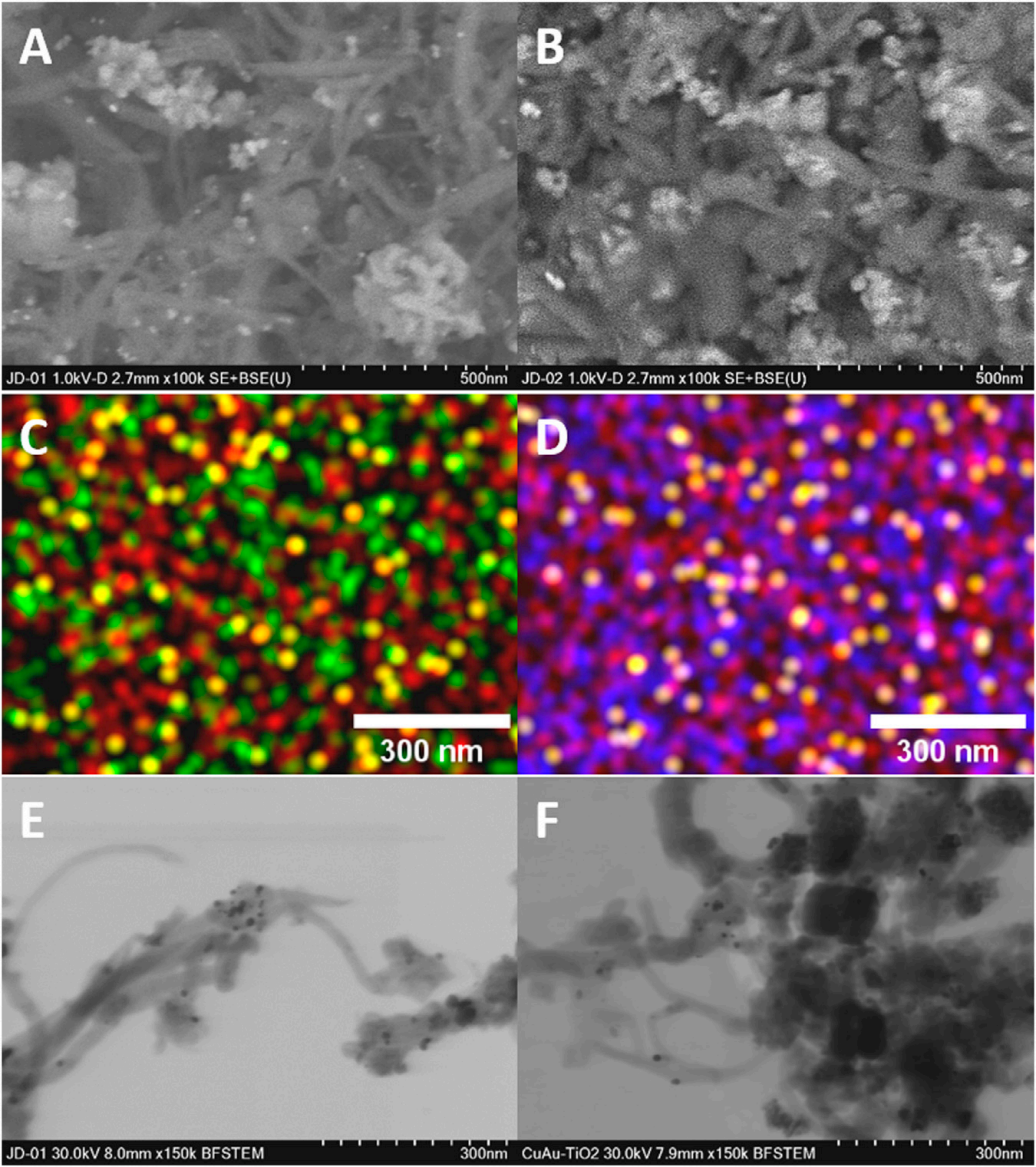
Images of Cu(18)Au(3)/MWCNT–CeO_2_(25) of **(A)** SEM, **(C)** EDS, and **(E)** STEM and images of Cu(18)Au(3)/MWCNT–TiO_2_(25) of **(B)** SEM, **(D)** EDS, and **(F)** STEM. In EDS maps, the colors represent different elements (yellow = Au, red = Cu, green = Ce, and blue = Ti).

The SEM images of the hybrid Cu(18)Au(3)/MWCNT–CeO_2_(25) and Cu(18)Au(3)/MWCNT–TiO_2_(25) catalysts (Figures 8A, B) show a homogenous composition with some agglomerates and a good integration of the components, mainly in the form of fibers and cumulus. CeO_2_ NPs are present as needle-like fibers with a more elongated and narrower morphology than the observed for TiO_2_ NPs. EDS mapping (Figures 8C, D) shows the interaction of the components with each other confirming the homogeneity of the materials. STEM images (Figures 8E, F) confirm that MWCNTs act as linkers among the different phases, supporting the mesoporous Cu_2_O particles and gold-coated Cu agglomerates in an irregular shape. Furthermore, in the case of the catalyst with the TiO_2_ support, Au spherical NPs interacting with TiO_2_ were also observed. Complementary images are presented in Supplementary Figures S2, S3.

##### 3.3.2.2 XPS

**FIGURE 9:**
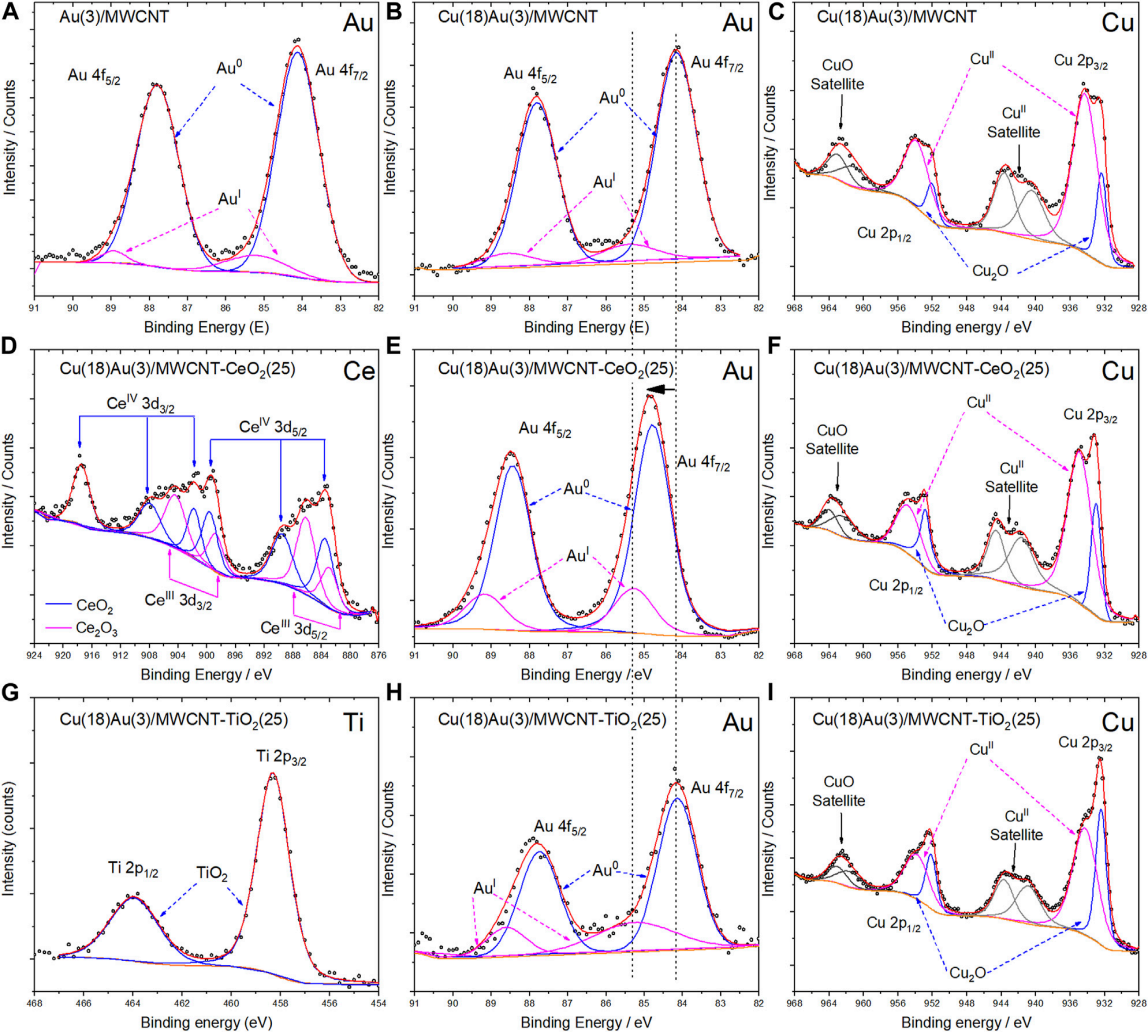
High-resolution XPS images for the Au(3)/MWCNT Au spectrum **(A)**; Cu(18)Au(3)/MWCNT **(B)** Au spectrum and **(C)** Cu spectrum; Cu(18)Au(3)/MWCNT/CeO_2_(25) **(D)** Ce spectrum, **(E)** Au spectrum, and **(F)** Cu spectrum; Cu(28)Au(3)/MWCNT/TiO_2_(25) **(G)** Ti spectrum, **(H)** Au spectrum, and **(I)** Cu spectrum. Black dots represent the experimental data, and the continuous lines represent the adjustment to the theoretical model.

XPS measurements were performed to study the chemical compositions of the surfaces for selected catalysts (Figure 9): Au(3)/MWCNT (Figure 9A), Cu(18)Au(3)/ MWCNT (Figures 9B, C), Cu(18)Au(3)/MWCNT–CeO_2_(25) (Figures 9D–F), and Cu(18)Au(3)/ MWCNT–TiO_2_(25) (Figure 9G–I). Figure 9A shows the Au-spectrum for the Au(3)/MWCNT catalyst, where Au^0^ is appreciated with a signal of *4f*_*5/2*_, with a binding energy of 84.2 eV (Wang X. et al., 2013). For the same region for Cu(18)Au(3)/MWCNT and Cu(18)Au(3)/MWCNT–TiO_2_(25) samples, the Au main signal is observed at the same binding energy (84.2 eV) (Figures 9B, H), while in the case of the Cu(18)Au(3)/MWCNT–CeO_2_(25) catalyst (Figure 9E), an important displacement to a higher binding energy (84.7 eV) takes place, indicating a strong interaction between Au NPs with CeO_2_ nanobars. Moreover, in the presence of CeO_2_ and TiO_2_, after deconvolution, a signal assigned to Au(I) is observed at 85.3 and 85.1 eV, respectively, indicating the stabilization of Au(I) sites, which are recognized to be highly active in heterogeneous catalysis. Figures 9C, F, I present the XPS spectra obtained in the region of Cu for Cu(18)Au(3)/MWCNT, Cu(18)Au(3)/MWCNT–CeO_2_(25), and Cu(18)Au(3)/MWCNT–TiO_2_(25) catalysts, respectively. In all spectra, the two main bands corresponding to the levels *2p*_*3/2*_ and *2p*_*1/2*_ located at 933 and 953 eV, respectively, are observed. The deconvolution of the signals indicated the presence of Cu_2_O and CuO, as previously observed by FTIR. The presence of satellite signals, located at 942 and 963 eV, confirmed the presence of CuO, probably formed by the oxidation of Cu_2_O (Wu et al., 2006; Sahai et al., 2016; Dan et al., 2018). For the catalyst containing CeO_2_, a slight displacement of around 0.6 eV is observed indicating an interaction between Cu and CeO_2_. On the other hand, no interactions between Cu and TiO_2_ are evident. Concerning the spectrum in the region of cerium (Figure 9D), two oxidation states at the surface (Ce_2_O_3_ and CeO_2_), typically observed in NPs, were identified by signals at 917.54 eV for Ce(IV) and 904.55 and 886.23 eV for Ce(III) (Yu et al., 2015; Li et al., 2018). Finally, in the case of titanium (Figure 9G) the characteristic TiO_2_ profile composed of the signal doublet at 458.30 and 463.93 eV corresponding to *2p*_*3/2*_ and *2p*_*1/2*_ levels, respectively, are observed (Sinatra et al., 2015). It is worth noticing that XPS results clearly indicate the influence of reducible CeO_2_ and TiO_2_ supports on the chemical environment of Au and Cu. Detailed information of the XPS analysis is presented in Supplementary Table S2.

##### 3.3.2.3 Cyclic voltammetry

**FIGURE 10:**
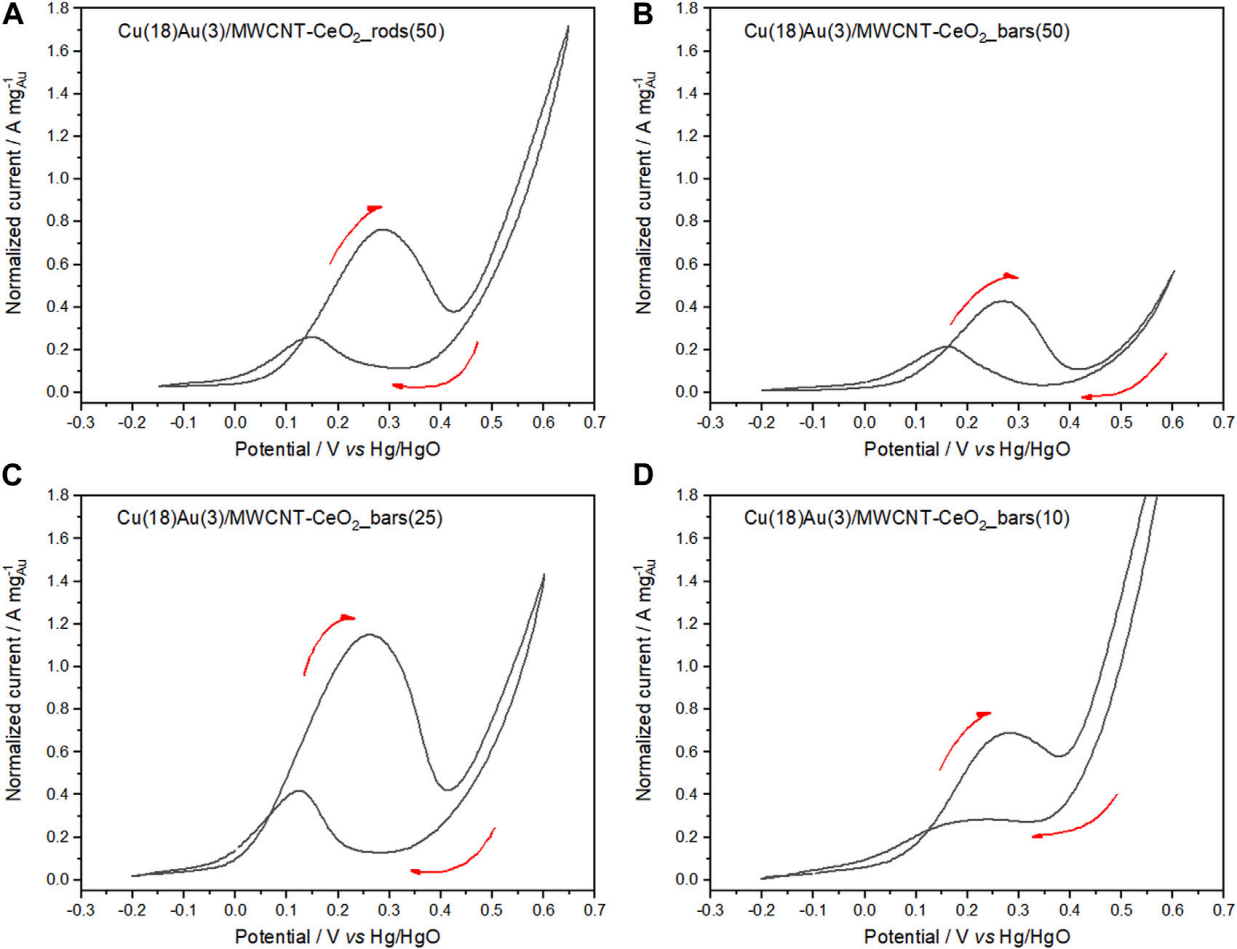
Cyclic voltammograms of Cu(18)Au(3)/MWCNT–CeO_2__rods (50) **(A)**; Cu(18)Au(3)/MWCNT–CeO_2__bars containing different CeO_2_ amounts: **(B)** 50, **(C)** 25, and **(D)** 10 wt% in 0.5 mol L^−1^ NaOH solution containing 0.1 mol L^−1^ glycerol (sweep rate of 50 mV s^−1^ at 25°C).

From the cyclic voltammograms presented in Figures 10A, B, the effect of the morphology of nanorods *vs.* nanobars is clearly observed and may be confirmed by the corresponding current integration ratio values of 3.54 and 2.02 for (A_f_/A_b_)_nanorods_ and (A_f_/A_b_)_nanobar_, respectively. In Figures 10C, D, the cyclic voltammograms of hybrid catalysts containing CeO_2_ with a nanobar morphology and varying amounts of the oxide (25 and 10 wt%) are shown. Comparing Figures 10B, C, the 25 wt%-containing bar catalyst exhibits a higher normalized peak current than the 50 wt% catalyst. This feature can be explained by the limiting conducting properties of CeO_2_ as compared to MWCNTs because as the amount of CeO_2_ decreased, the amount of MWCNTs increased during the preparation procedure. In the cases of 50 and 25 wt%, forward and backward sweeps present similar profiles. However, a further decrease in the amount of CeO_2_ to 10 wt% (Figure 10D) led to a reduction in forward and backward peak currents. In addition, according to the current integration ratios for 50, 25, and 10 wt% CeO_2_-nanobar catalysts of 2.02, 4.07, and 4.59, respectively, the catalyst with 10 wt% presents the best reducibility of the surface (Lai et al., 2021). Nevertheless, considering the normalized peak currents obtained for the set of catalysts shown in Figure 10, the 25 wt% bar catalyst performs the best in the GlyEOR. The results obtained with 50 wt% CeO_2_ nanorods are quite similar to those obtained with 25 wt% CeO_2_ nanobars, despite the longer and thinner structure of the nanorods that allow a higher amount of the more active crystal facets (100). This similarity is observed primarily because both morphologies expose the same active crystal facets, (110) and (100) (Zhang et al., 2014).

**FIGURE 11:**
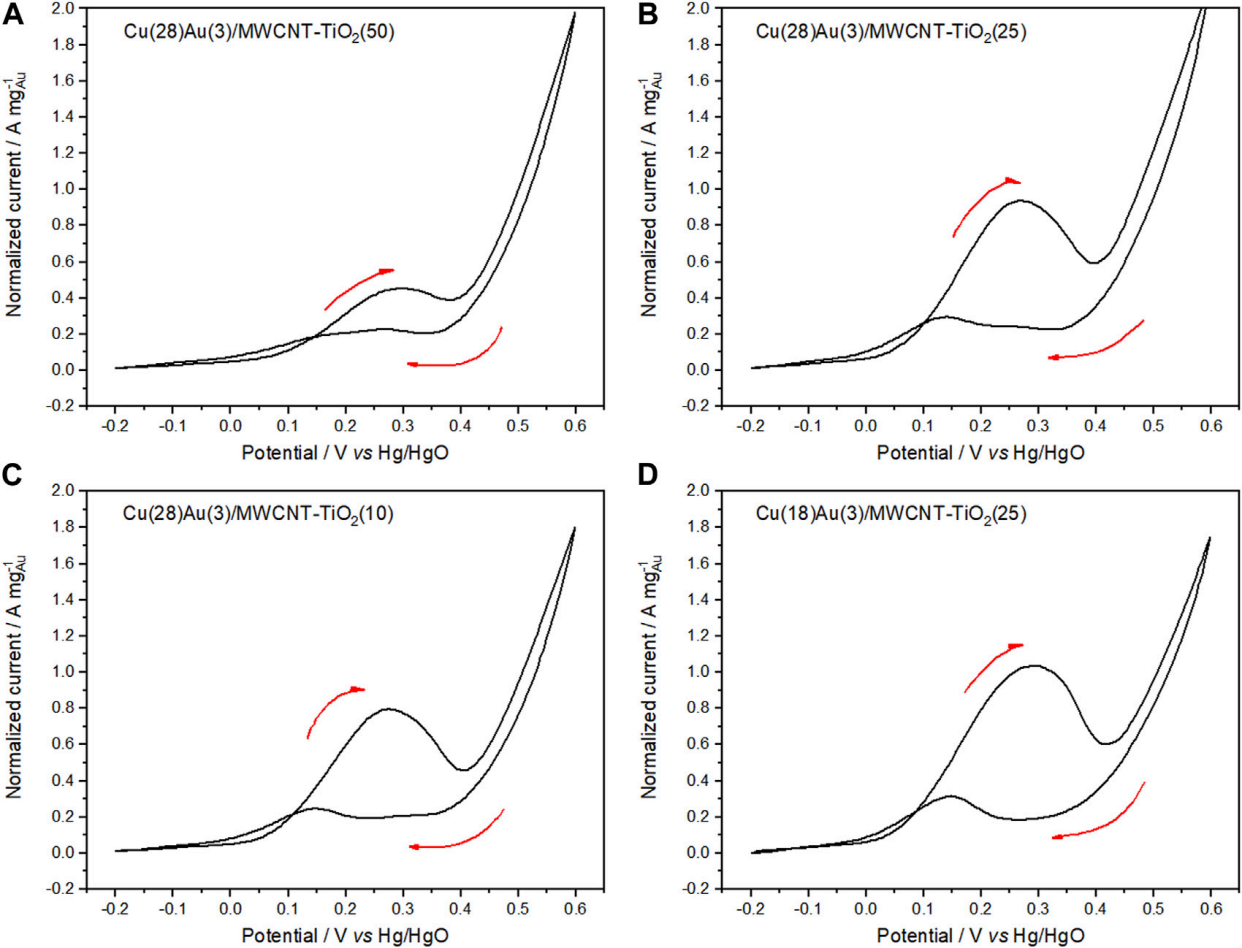
Cyclic voltammograms of Cu(28)Au(3)/MWCNT–TiO_2_ **(A)** 50, **(B)** 25, **(C)** 10 wt%, and Cu(18)Au(3)/MWCNT–TiO_2_ **(D)** 25 wt% in 0.5 mol L^−1^ NaOH solution containing 0.1 mol L^−1^ glycerol (sweep rate of 50 mV s^−1^ at 25°C).

CV of hybrid catalysts Cu(28)Au(3)/MWCNT–TiO_2_ with varying amounts of TiO_2_ as a support (figures 11A–C) was performed under the same conditions as in the case of CeO_2_. Contrary to the case with CeO_2_, no significant changes were observed when the amount of TiO_2_ increased from 10 to 25 wt% (Figures 11B, C). An increase of 50 wt% in the amount of TiO_2_ causes a diminishment in the normalized peak current probably because of the lower conductivity of TiO_2_ compared with that of MWCNTs (Figure 11A). The effect of the amount of Cu in these catalysts can be observed by comparing Figures 11B, D, where the amount Cu is diminished to 18 wt%, while keeping the amount of Au and TiO_2_ constant. No significant differences are made evident in the normalized peak current. However, the current peak ratio increases as the amount of Cu increases, becoming evident. Thus, concluding that the catalyst that performs the best is Cu(28)Au(3)/MWCNT–TiO_2_(25). Table 4 summarizes the electrochemical parameters of the voltammograms for the studied catalyst.

**TABLE 4:** Electrochemical parameters of the fifth cycle for bimetallic hybrid catalysts.

Catalyst	Forward scan			Backward scan			A_pf_/A_pb_
	E_onset_ (mV)	E_pf_ (mV)	I_pf_ (A mg^−1^_Au_)	E_onset_ (mV)	E_pb_ (mV)	I_pb_ (A mg^−1^_Au_)	
Cu(18)Au(3)/MWCNT–CeO_2_ nanorods (50)	9	287	0.763	304	148	0.261	3.54
Cu(18)Au(3)/MWCNT–CeO_2_ nanobars (10)	−13	280	0.690	318	240	0.283	4.59
Cu(18)Au(3)/MWCNT–CeO_2_ nanobars (25)	−60	261	1.150	267	126	0.418	4.07
Cu(18)Au(3)/MWCNT–CeO_2_ nanobars (50)	−4	271	0.428	332	161	0.216	2.02
Cu(28)Au(3)/MWCNT–TiO_2_ (10)	−7	278	0.794	235	147	0.245	5.45
Cu(28)Au(3)/MWCNT–TiO_2_ (25)	−11	282	0.937	265	147	0.286	3.92
Cu(18)Au(3)/MWCNT–TiO_2_ (25)	−16	285	1.045	292	146	0.319	4.47
Cu(28)Au(3)/MWCNT–TiO_2_ (50)	−4	298	0.364	335	268	0.181	2.00

## 4 Conclusion

Cu and Au mono- and bimetallic NPs were supported on MWCNTs for use as catalysts in the GlyEOR in alkaline media. Owing to the intermatrix synthesis procedure, a Cu(I) oxidation state was achieved and further stabilized as Cu_2_O coated with Au(0) by galvanic displacement, as demonstrated by FTIR, XRD, and XPS characterizations. The high energy of the Cu(I) electronic state and Au(I) sites found in the bimetallic catalyst explains the enhanced catalytic activity observed for the GlyEOR compared to that of the Au(0) monometallic catalyst. An increase in the amount of Cu_2_O in the Cu(9,18,28)Au(3)/MWCNT catalyst increased the catalytic activity owing to the synergy between Au and Cu. With the aim of enhancing the reducibility of the Au surface and simultaneously reducing the susceptibility of Au to poisoning species such as CO, a strategy involving the production of hybrid catalysts containing reducible oxides with high oxophilicity as supports was applied. For the introduction of CeO_2_ or TiO_2_ as NPs, the advantage of an easier, more eco-friendly, less time-consuming synthetic hydrothermal microwave-assisted methodology was developed allowing a higher yield. Despite that for CeO_2_, the nanorod morphology has been recognized as the most active for several applications, under optimized conditions; the Cu–Au/MWCNT/CeO_2_-nanobar morphology proved to have comparable performance to CeO_2_ nanorods (50 wt%). This can be explained by the fact that both morphologies exhibit the same active crystal facets. Considering that high amounts of CeO_2_ nanobars limit the conductivity of the material, and thus, the catalytic activity, an optimization of the composition of the hybrid catalyst, allowed us to conclude that the best performance was achieved with the catalyst Cu(28)Au(3)/MWCNT/CeO_2_(25)_nanobars. Furthermore, the catalyst Cu(28)Au(3)/MWCNT/TiO_2_(25) demonstrates an improved behavior over Cu(28)Au(3)/MWCNT and Cu(28)Au(3)/MWCNT/TiO_2_(50) but lower catalytic activity than Cu(28)Au(3)/MWCNT/CeO_2_(25) nanobars, as the latter presents the redox pair Ce(IV)/Ce(III) on its surface. In contrast, a better performance of the catalysts containing TiO_2_ may be related to a more homogeneous integration of the components and the interaction between TiO_2_ and Au NPs evidenced by the increase in the A_pf_/A_pb_ ratio. In summary, these results reveal that, for the as-obtained hybrid catalysts, the reducible oxides play the role of oxophilic species and facilitate the dispersion and stabilization of Au and Cu species. Thus, the overall effect derives in a higher catalytic activity in the GlyEOR. Further studies are essential to determine the selectivity and stability of hybrid catalysts.

## Data Availability

The original contributions presented in the study are included in the article/Supplementary Material; further inquiries can be directed to the corresponding author
