# Supplementary material for: Enhanced performance of glycerol electro-oxidation in alkaline media using bimetallic Au–Cu NPs supported by MWCNTs and reducible metal oxides
Source: Front Chem. 2023 Jul 3;11:1165303. doi: 10.3389/fchem.2023.1165303 (PMC10351873; doi:10.3389/fchem.2023.1165303)
Supplement: Supplementary file 1 [file DataSheet1.PDF]

Enhanced Performance of Glycerol Electro-Oxidation in Alkaline Media Using Bimetallic Au-Cu NPs Supported on MWCNT and Reducible Metal Oxides

Josefina de Gyves[1], Luis G. Molina-Ruiz, Erik Rutz-López, Alejandro Gutiérrez-Sánchez, Ana Lilia Ocampo[1], Nadia M. Munguía-Acevedo[1], Frida Peña-Medina and Vicente Esquivel-Peña[1]*

[1] Facultad de Química, Departamento de Química Analítica, Universidad Nacional Autónoma de México, 04510. Ciudad de México. México.

SUPPORTING INFORMATION

Table S1. FTIR-spectra band assignments of the functionalized MWCNTs after the addition of $NaBH_4$, and in the presence of AuNPs, CuNPs, and Cu-AuNPs.

Material	Wavenumber (cm^{-1})	Assigment
**MWCNT + $NaBH_4$**	3480-3430	O-H stretching
	3280-3220	N-H stretching
	1725	C=O elongation
	1575	C=C stretching
	1380	N-O stretching
	1200	C-O stretching from alcohols
	1095	C-O stretching from alcohols
**Au(3)/MWCNT**	3434	O-H stretching
	3220	N-H stretching
	1725	C=O elongation
	1630	C=C stretching
	1200	C-O stretching from alcohols
	1015	C-O stretching from alcohols
**Cu(20)/MWCNT**	3434	O-H stretching
	3220	N-H stretching
	1574	C=C stretching
	1382	C-O deformation
	1196	C-O stretching from alcohols
	630	Cu_2O
**Cu(18)Au(3)/MWCNT**	3480-3150	O-H stretching
	1574	C=C stretching
	1156	C-O stretching from alcohols
	630	Cu_2O

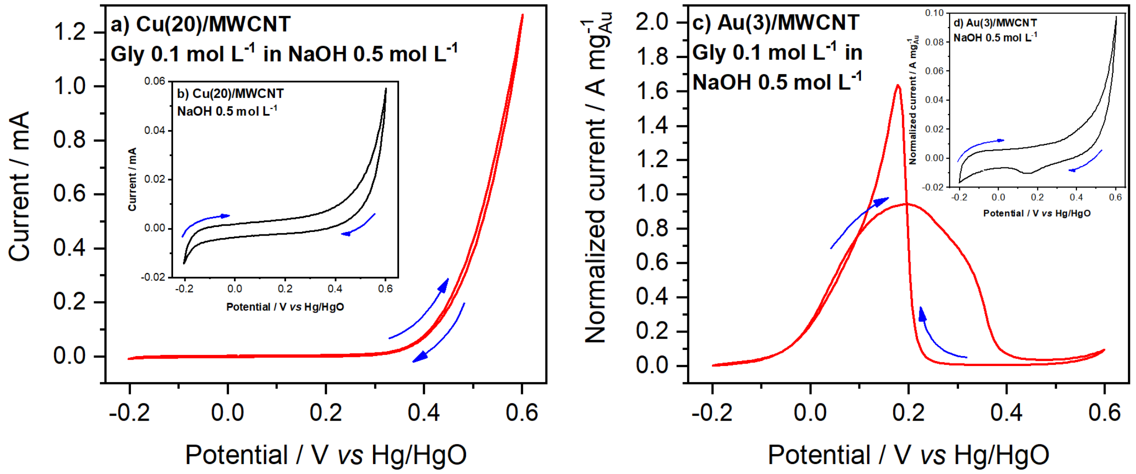

Figure S1. Cyclic voltammogram obtained for the Cu(20)/MWCNT catalyst in: (a) 0.5 mol L^{-1} NaOH and (b) 0.1 mol L^{-1} glycerol in 0.5 mol L^{-1} NaOH, and Au(3)/MWCNT catalyst in: (c) 0.5 mol L^{-1} NaOH and (d) 0.1 mol L^{-1} glycerol in 0.5 mol L^{-1} NaOH

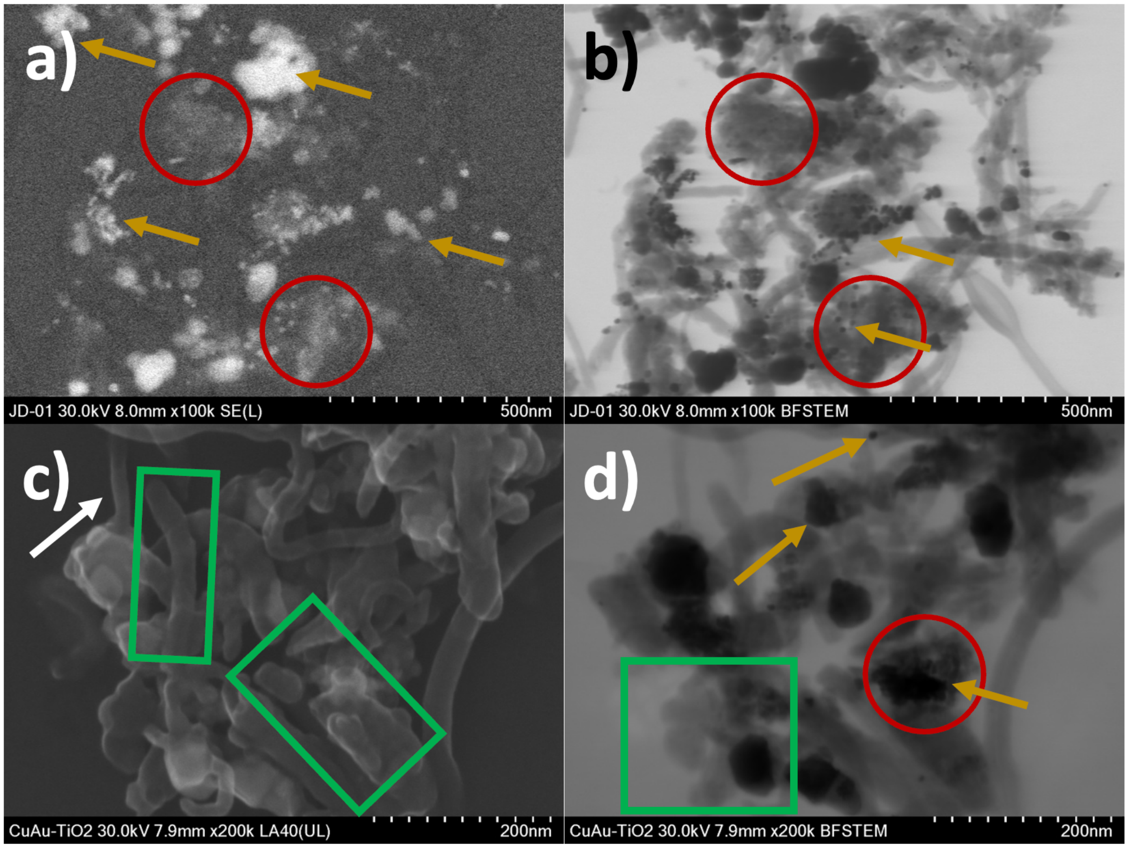

Figure S2. STEM **i**mages of Cu(18)Au(3)/MWCNT-CeO_2(25) and Cu(18)Au(3)/MWCNT-TiO_2(25). Images showing the contrast in morphology between Cu, Au, and MO_2 NPs. Red

circles represent the Cu matrix, green rectangles indicate TiO_2 NPs, golden arrows point to AuNPs and white arrows point to MWCNT matrix.

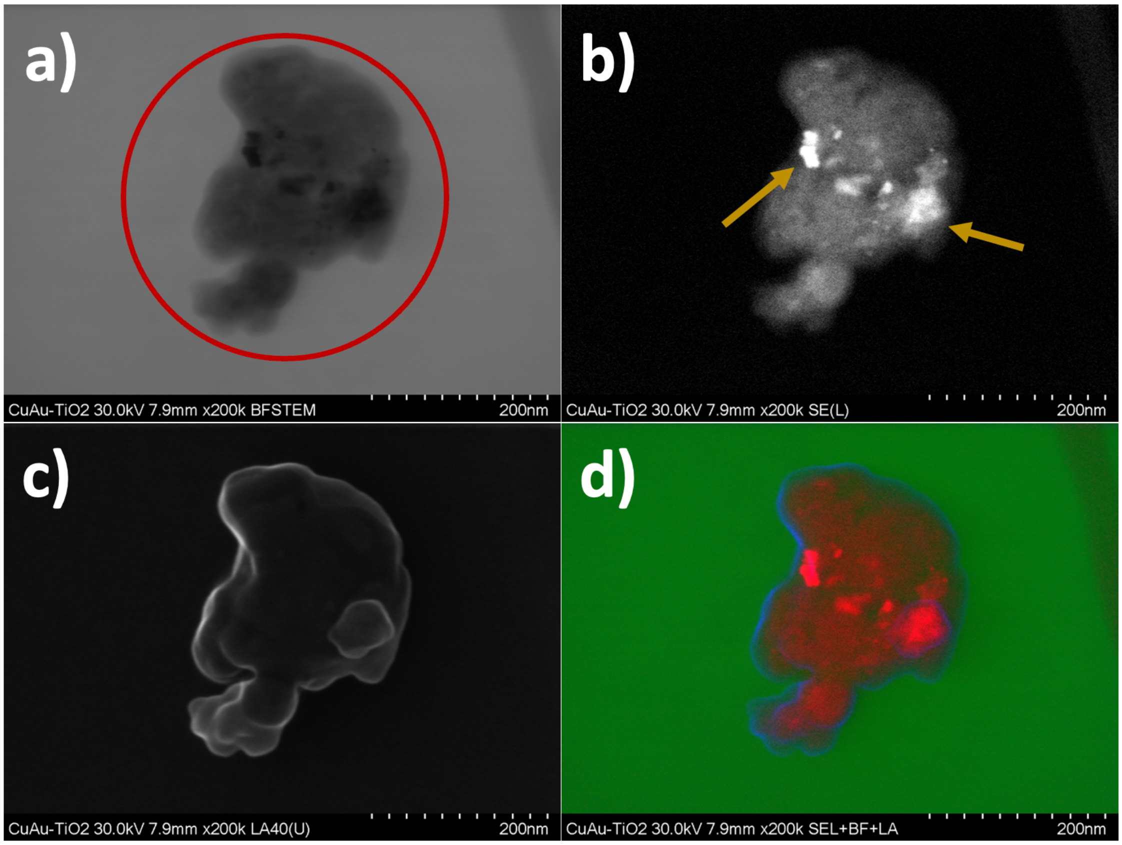

Figure S3. STEM **i**mages of Cu(18)Au(3)/MWCNT-TiO_2(25). Images showing the contrast in morphology between the Cu_2O agglomerates with AuNPs on their surface. Red circle represents the Cu matrix and golden arrows point to AuNPs

Table S2. Surface chemical composition obtained by XPS

Sample	Element	Core level	B.E. (eV)	Atomic percentage
Au(3)/MWCNT	Au(0)	$4f_{5/2}$	84.12	90.5%
	Au(0)	$4f_{5/2}$	85.22	9.5%
Cu(18)Au(3)/MWCNT	Au(0)	$4f_{5/2}$	84.16	90.3%
	Au(0)	$4f_{5/2}$	85.40	9.7%
	Cu(I)	$2p_{3/2}$	932.34	19.4%
	Cu(II)	$2p_{3/2}$	934.35	80.6%
Cu(18)Au(3)/MWCNT-CeO2(25)	Au(0)	$4f_{5/2}$	84.79	79.7%
	Au(I)	$4f_{5/2}$	85.30	20.3%
	Cu(I)	$2p_{3/2}$	933.04	22.1%
	Cu(II)	$2p_{3/2}$	935.09	77.9%
	Ce(III)	$3d_{5/2}$	882.90	32.5%
	Ce(IV)	$3d_{5/2}$	883.64	67.5%
Cu(18)Au(3)/MWCNT-TiO2(25)	Au(0)	$4f_{5/2}$	84.15	82.9%
	Au(I)	$4f_{5/2}$	85.12	17.1%
	Cu(I)	$2p_{3/2}$	932.41	44.4%
	Cu(II)	$2p_{3/2}$	934.38	55.6%
	Ti(IV)	$2p_{3/2}$	458.33	100.0%
Cu(28)Au(3)/MWCNT-TiO2(25)	Au(0)	$4f_{5/2}$	84.13	66.8%
	Au(I)	$4f_{5/2}$	85.10	33.2%
	Cu(I)	$2p_{3/2}$	932.46	36.1%
	Cu(II)	$2p_{3/2}$	934.46	63.9%
	Ti(IV)	$2p_{3/2}$	458.33	100.0%
